# Supplementary material for: Mapping the ecological networks of microbial communities
Source: Nat Commun. 2017 Dec 11;8:2042. doi: 10.1038/s41467-017-02090-2 (PMC5725606; doi:10.1038/s41467-017-02090-2)
Supplement: Supplementary file 1 — Supplementary Information [file 41467_2017_2090_MOESM1_ESM.pdf]

MAPPING THE ECOLOGICAL NETWORKS OF MICROBIAL COMMUNITIES

Supplementary Note 1: Theoretical basis for inferring ecological interactions **2**
Notation 2
Preliminaries 2
Inferring the zero-pattern 3
Inferring the sign-pattern 4
Supplementary Note 2: Brute-force and heuristic algorithms **9**
Brute-force algorithm 9
Inference using the heuristic algorithm 10
Supplementary Note 3: Inferring the topology of ecological networks **13**
Supplementary Note 4: Inferring the ecological interaction types **15**
Supplementary Note 5: Inferring interaction strengths with GLV dynamics **16**
A condition for detecting GLV dynamics. 16
Inference of interaction strengths and intrinsic growth rates 16
Applying the Knockoff filter to control the false discovery rate 16
Blinded inference of interaction strengths by assuming GLV dynamics. 17
Supplementary Note 6: Real datasets **18**
A synthetic microbial community of eight soil bacteria. 18
A synthetic community of maize roots with seven bacterial species. 18
A synthetic microbial community of two cross-feeding partners. 18
A synthetic community of 14 auxotrophic *Escherichia coli* strains 19
Supplementary Note 7: Relationship to existing notions of inter-taxa interactions . . **20**
Supplementary References **21**
Supplementary Figures **22**

Supplementary Note 1: Theoretical basis for inferring ecological interactions

Here we formulate and prove two theorems (Theorems 1 and 2) that characterize the conditions for inferring the presence/absence or type (positive, negative or neutral) of ecological interactions in a microbial community using steady-state data. These theorems provide the basis for the inference methods described in Supplementary Note .

Notation. We use bold letters like $\boldsymbol{x}$ to denote vectors, and capitals like J to denote matrices. The i-th element of the vector $\boldsymbol{x}$ is denoted by x_i. Similarly, $\boldsymbol{J}_i$ denotes the i-th row of matrix J, and J_{ij} denotes the (i,j)-th element of matrix J. For a matrix $J \in \mathbb{R}^{N\times N}$, we denote by $S = (s_{ij}) = \mathsf{sign}(J) \in \{-1, 0, 1\}^{N\times N}$ its sign-pattern, where $s_{ij} = \mathsf{sign}(J_{ij})$. Similarly, we denote by $Z = (z_{ij}) \in \{0, 1\}^{N\times N}$ its zero-pattern, where $z_{ij} = |s_{ij}|$.

Preliminaries. Consider a microbial community of N different taxa. Let $x_i(t)$ denote the absolute abundance of taxon i at time t. Suppose the temporal evolution of the taxa abundances are described by a generic population dynamics model taking the form of a set of ordinary differential equations (ODEs):

$$\frac{dx_i(t)}{dt} := \dot{x}_i(t) = x_i(t) f_i\left(\boldsymbol{x}(t)\right), \quad i = 1, \cdots, N, \tag{S1}$$

where $\boldsymbol{x} = (x_1, \cdots, x_N)^\intercal \in \mathbb{R}^N$ is the state vector and $f_i : \mathbb{R}^N \to \mathbb{R}$, $i = 1, \cdots, N$, are some non-zero *meromorphic* functions —that is, the quotient of two analytical functions of $\boldsymbol{x}$.

Remark 1. Typical examples of meromorphic functions are in the form of the quotient of two polynomials. By specifying these meromorphic functions, system (S1) can take the form of many classical population dynamics models [1, 2, 3, 4, 5, 6]. The assumption that all $f_i(x)$'s are some non-zero meromorphic functions has a useful consequence, since meromorphic functions have the *generic* properties inherited from analytic functions [7]. This implies, for example, that since the f_i's are not identically zero, they can be zero only on a zero-measure set of their domain $\mathbb{R}^N$.

A steady-state dataset $\mathcal{X}$ is a collection of N-dimensional vectors $\boldsymbol{x} \in \mathbb{R}^N$ corresponding to the measured equilibria of Eq. (S1). Each element of $\mathcal{X}$ is called a steady-state sample, or just a sample. We will denote a sample as $\boldsymbol{x}^I \in \mathbb{R}^N$, where its taxon *index set* $I \in \mathcal{I}$ determines which taxa are present. Here $\mathcal{I} = 2^{\{1,\cdots,N\}}$ is the set of all possible subsets of $\{1, \cdots, N\}$. For example, $\boldsymbol{x}^{\{1,2\}} \in \mathbb{R}^3$ is a sample of a community with three taxa, and in this sample only taxon 1 and taxon 2 are present.

Consider now the subset $\mathcal{X}_i \subseteq \mathcal{X}$ of all samples containing taxon i, so that $f_i(\boldsymbol{x}) = 0$ for all $\boldsymbol{x} \in \mathcal{X}_i$. Then, for any two samples $\boldsymbol{x}^I, \boldsymbol{x}^K \in \mathcal{X}_i$, applying the mean value theorem for multi-variable functions, we obtain

$$f_i(\boldsymbol{x}^I) - f_i(\boldsymbol{x}^K) = \left(\int_0^1 \frac{\partial f_i(\boldsymbol{x}^I + \sigma(\boldsymbol{x}^K - \boldsymbol{x}^I))}{\partial \boldsymbol{x}} d\sigma \right) \cdot (\boldsymbol{x}^I - \boldsymbol{x}^K) = 0, \tag{S2}$$

where '$\cdot$' denotes the inner product between vectors in $\mathbb{R}^N$. Let $\boldsymbol{J}_i(\boldsymbol{x}) = \partial f_i(\boldsymbol{x})/\partial \boldsymbol{x} \in \mathbb{R}^N$ be the i-th row of the Jacobian matrix $J(\boldsymbol{x}) = (J_{ij}(\boldsymbol{x})) = (\partial f_i(\boldsymbol{x})/\partial x_j)$ and let us introduce the notation

$$\int_{L_{\boldsymbol{x}^I, \boldsymbol{x}^K}} \boldsymbol{J}_i := \int_0^1 \boldsymbol{J}_i\big(\boldsymbol{x}^I + \sigma(\boldsymbol{x}^K - \boldsymbol{x}^I)\big) d\sigma,$$

where $L_{\boldsymbol{x}^I, \boldsymbol{x}^K}$ denotes the line segment joining the points $\boldsymbol{x}^I$ and $\boldsymbol{x}^K$ in $\mathbb{R}^N$. With this notation, Eq. (S2) can be rewritten more compactly as

$$\left(\int_{L_{\boldsymbol{x}^I, \boldsymbol{x}^K}} \boldsymbol{J}_i \right) \cdot (\boldsymbol{x}^I - \boldsymbol{x}^K) = 0, \quad \forall \boldsymbol{x}^I, \boldsymbol{x}^K \in \mathcal{X}_i. \tag{S3}$$

The above equation implies that the difference of any two samples $\{\boldsymbol{x}^I, \boldsymbol{x}^K\}$ sharing taxon i constrains the integral of $\boldsymbol{J}_i$ over the line segment joining them $\boldsymbol{x}^I - \boldsymbol{x}^K$.

In this work, we consider that the ecological interactions in a microbial community are encoded in the Jacobian matrix $J \in \mathbb{R}^{N\times N}$ of its population dynamics. More precisely, we assume that the j-th taxon directly impacts the i-th one iff the function $J_{ij}(\boldsymbol{x}) \not\equiv 0$. Notice that this condition is well defined because $J_{ij}(\boldsymbol{x})$ is a meromorphic function. Further, an ecological interaction is inhibitory iff $J_{ij}(\boldsymbol{x}) < 0$ and excitatory iff $J_{ij}(\boldsymbol{x}) > 0$.

Inferring the absence or presence of interactions is equivalent to inferring the zero-pattern of the Jacobian matrix, recovering the topology of the ecological network underlying the microbial community. Furthermore, inferring the type of interactions (inhibitory, excitatory or null) is equivalent to inferring the sign-pattern of the Jacobian matrix. Analyzing the implications of Eq. (S3) will be the basis for inferring these two properties of the Jacobian matrix from the steady-state samples $\mathcal{X}$, as we next show.

Inferring the zero-pattern. Let $\int_{L_{\boldsymbol{x}^I,\boldsymbol{x}^K}} J_{ij}$ denote the j-th entry of the vector $\left(\int_{L_{\boldsymbol{x}^I,\boldsymbol{x}^K}} \boldsymbol{J}_i\right) \in \mathbb{R}^N$. To infer the zero-pattern of the Jacobian matrix, we make the following assumption:

Assumption 1. The condition $\int_{L_{\boldsymbol{x}^I,\boldsymbol{x}^K}} J_{ij} \equiv 0$ holds if and only if $J_{ij} \equiv 0$ for all $i, j = 1, \cdots, N$.

Remark 2.

a. Assumption 1 is a *necessary condition* to recover the zero-pattern of $\boldsymbol{J}_i$, $i = 1, \cdots, N$, from steady-state samples $\mathcal{X}$, regardless of the algorithm used for the inference. Indeed, if $\int_{L_{\boldsymbol{x}^I,\boldsymbol{x}^K}} J_{ij} \equiv 0$ but $J_{ij} \not\equiv 0$, then it is impossible to distinguish from the samples $\mathcal{X}$ if either $J_{ij} \equiv 0$ or $J_{ij} \not\equiv 0$, as both conditions would satisfy Eq. (S3).
b. Assumption 1 is generically satisfied for most functions $f_i(x)$ used in population dynamics models. More precisely, notice how the condition $0 \equiv \int_{L_{\boldsymbol{x}^I,\boldsymbol{x}^K}} J_{ij} = \int_0^1 J_{ij}(\boldsymbol{x}^I + \sigma(\boldsymbol{x}^K - \boldsymbol{x}^I))d\sigma$ requires that the positive and negative areas below $J_{ij}(\boldsymbol{x}^I + \sigma(\boldsymbol{x}^K - \boldsymbol{x}^I))$ cancel exactly when plotted as a function of σ. Such condition is not *generic*. This means that if the above equation holds for some particular f_i and its corresponding J_{ij}, then there exists an infinitesimal deformation $\tilde{f}_i$ of f_i such that the areas of the corresponding $\tilde{J}_{ij}$ as a function of σ do not cancel out anymore.

For each sample pair $\{\boldsymbol{x}^I, \boldsymbol{x}^K\}$, let's denote by $\mathcal{Z}_{I,K} \subseteq \{0,1\}^N$ the set of zero-patterns of all vectors orthogonal to $\boldsymbol{x}^I - \boldsymbol{x}^K$. Then we obtain the following result:

Theorem 1. Let $\boldsymbol{Z}_i \in \{0,1\}^N$ be the zero-pattern of $\boldsymbol{J}_i$. Then, under Assumption 1, we have that

$$\boldsymbol{Z}_i \in \hat{\mathcal{Z}}_i := \bigcap_{\boldsymbol{x}^I,\boldsymbol{x}^K \in \mathcal{X}_i} \mathcal{Z}_{I,K}.$$

Proof. From Assumption 1, we conclude that $\boldsymbol{Z}_i$ and the zero-pattern of $\int_{I,K} \boldsymbol{J}_i$ are identical for all $x^I, x^J \in \mathcal{X}_i$. Then Eq. (S3) implies that the $\boldsymbol{Z}_i \in \mathcal{Z}_{I,K}$ for all $\boldsymbol{x}^I, \boldsymbol{x}^J \in \mathcal{X}_i$. This directly implies that $\boldsymbol{Z}_i \in \hat{\mathcal{Z}}_i$. □

Remark 3.

a. Note that $\hat{\mathcal{Z}}_i$ will always contain at least two elements: a trivial one $\boldsymbol{0} \in \mathbb{R}^N$ and a non-trivial one $\hat{\boldsymbol{z}}_i \in \{0,1\}^N$. Therefore, to distinguish which of these two zero-patterns is the true zero-pattern of $\boldsymbol{J}_i$, it is necessary to a-priori know the existence of at least one nonzero interaction.
b. Theorem 1 together with Remark 3.a provide the basis of a computational method to infer the zero-pattern of the Jacobian matrix, since the sets $\mathcal{Z}_{I,K}$ can be computed from the steady-state data $\mathcal{X}$.

Example 1. Consider a toy model with two taxa:

$$\dot{x}_1 = x_1(0.5 - x_1 + 0.1x_2), \dot{x}_2 = x_2(x_2 - 0.6)(0.2 - x_2),$$

where the Jacobian matrix is

$$J = \begin{pmatrix} -1 & 0.1 \\ 0 & 0.8 - x_2 \end{pmatrix}.$$

Note that the sign of J_{22} depends on the value of x_2. Supplementary Figure 1b shows that J_{22} indeed changes its sign from positive to negative during the growth process (Supplementary Figure 1a).

In the absence of measurement noise, we can successfully infer the zero-pattern of J (Supplementary Figure 1c,d). Supplementary Figure 1c shows that, according to the position of $\boldsymbol{x}^{\{1,2\}} - \boldsymbol{x}^{\{1\}}$, the green line that is orthogonal to the red line cannot yield a zero entry for $\boldsymbol{J}_1$, implying that $J_{11} \neq 0$ and $J_{12} \neq 0$. This is consistent with the ground truth. Supplementary Figure 1d shows that for $\boldsymbol{J}_2$, the green line that is orthogonal to the blue line exactly locates on the axis of x_2, implying that $J_{21} = 0$ and $J_{22} \neq 0$, which is also consistent with the ground truth.

In the presence of measurement noise, the angle between the x_1-axis (or the x_2-axis) and the green line can be used to determine if $J_{ij} = 0$ or not (Supplementary Figure 1e,f). For example, when the noise level (η) is 0.1, in Supplementary Figure 1e the angle between the x_1-axis and the green line is large enough and we can safely conclude that $J_{12} \neq 0$. By contrast, the green line deviates only slightly from the x_2-axis and these deviations are randomly distributed and have zero mean (Supplementary Figure 1f). Therefore, we can choose a threshold value θ such that if the absolute value of the average deviation angle over different measurements is smaller than θ, we conclude that $J_{21} = 0$. Notice that this method will infer very weak interactions as zero, but it still offers a pragmatic approach to infer strong interactions.

Inferring the sign-pattern. In order to infer the sign-pattern, we assume:

Assumption 2. The nature of ecological interactions (i.e., parasitism, commensalism, mutualism, amensalism or competition) between any two taxa does not vary over the collected steady-state samples.

Note that Assumption 2 is actually necessary to infer the ecological interaction types. If those interaction types vary from samples to samples, then the inference becomes an ill-defined problem because we have a "moving target" and different subsets of steady-state samples will offer different answers on the interaction types.

Remark 4. Assumption 2 has the following consequences:

a. For $\boldsymbol{x}$ in the positive orthant of $\mathbb{R}^N$, each element $J_{ij}(\boldsymbol{x})$ of the vector $\boldsymbol{J}_i(\boldsymbol{x})$ is either uniformly negative, uniformly zero or uniformly positive. Indeed, if and only if this condition is satisfied, the sign-pattern of the Jacobian, given by $S = (s_{ij}) = (\mathsf{sign}(J_{ij}(\boldsymbol{x}))) \in \{-, 0, +\}^{N \times N}$, is constant.
b. The sign-pattern of the vectors $\int_{L_{\boldsymbol{x}^I,\boldsymbol{x}^K}} \boldsymbol{J}_i$ is the same for all $\boldsymbol{x}^I, \boldsymbol{x}^K \in \mathcal{X}_i$, and it coincides with the sign-pattern of $\boldsymbol{J}_i$.

With Assumption 2, next we show that the vector $(\boldsymbol{x}^I - \boldsymbol{x}^K)$ constrains enough the possible sign-pattern of the vector $\boldsymbol{J}_i$ so that we can infer it. Let us associate each orthant of $\mathbb{R}^N$ with its corresponding sign-pattern, that is, a vector in $\{-, 0, +\}^N$. There are exactly 3^N vectors in $\{-, 0, +\}^N$.

Example 2. $\{-, 0, +\}^2$ has 9 sign-patterns:

$$\{(-,-), (-,0), (-,+), (0,-), (0,0), (0,+), (+,-), (+,0), (+,+)\}.$$

And $\{-1, 0, 1\}^3$ has 27 sign-patterns.

In order to show how the vector $(\boldsymbol{x}^I - \boldsymbol{x}^K)$ constrains the sign-pattern of $\boldsymbol{J}_i$, we start by discussing the following elementary example:

Example 3. Consider $N = 2$ and three steady-state samples $\boldsymbol{x}^{\{1\}}$, $\boldsymbol{x}^{\{2\}}$ and $\boldsymbol{x}^{\{1,2\}}$. From Eq. (S3), we obtain:

$$\left(\int_{L_{\boldsymbol{x}^{\{1,2\}},\boldsymbol{x}^{\{1\}}}} \boldsymbol{J}_1 d\sigma\right) \cdot (\boldsymbol{x}^{\{1,2\}} - \boldsymbol{x}^{\{1\}}) = 0,$$

$$\left(\int_{L_{\boldsymbol{x}^{\{1,2\}},\boldsymbol{x}^{\{2\}}}} \boldsymbol{J}_2 d\sigma\right) \cdot (\boldsymbol{x}^{\{1,2\}} - \boldsymbol{x}^{\{2\}}) = 0.$$

These equations imply that $\int_{L_{\{1,2\},\{1\}}} \boldsymbol{J}_1 d\sigma$ is orthogonal to the line $L_{\{1,2\},\{1\}}$, and that $\int_{L_{\{1,2\},\{2\}}} \boldsymbol{J}_2 d\sigma$ is orthogonal to the line $L_{\{1,2\},\{2\}}$. Further, as discussed in Remark 4.b, recall that Assumption 2 implies $\mathsf{sign}(\int \boldsymbol{J}_i) = \mathsf{sign}(\boldsymbol{J}_i)$. Hence, $\mathsf{sign}(\boldsymbol{J}_i)$ is one of the sign-patterns corresponding to the line orthogonal to $L_{\{i,2\},\{i\}}$, see Supplementary Figure 2.

As a concrete example showing how the above equations can be used to infer the sign-pattern of the Jacobian matrix, consider the following ecological dynamics with the so-called Holling Type II functional response [2]:

$$\text{(S4)} \qquad \dot{x}_1 = x_1\left(r_1 + a_{11}x_1 + a_{12}\frac{x_2}{0.1h + x_2}\right), \quad \dot{x}_2 = x_2\left(r_2 + a_{21}\frac{x_1}{h + x_1} + a_{22}x_2\right).$$

Here $A = (a_{ij}) \in \mathbb{R}^{2\times 2}$, $r = (10, 5)^\intercal \in \mathbb{R}^2$ and $h = 1$ are parameters. Importantly, notice that $\mathsf{sign}(J) = \mathsf{sign}(A)$.

Next we focus on inferring $\mathsf{sign}(\boldsymbol{J}_1)$, as the same procedure can be applied to infer $\mathsf{sign}(\boldsymbol{J}_2)$. For illustration, we choose $a_{21} = -1, a_{22} = -1$ and then consider two cases for the remaining two parameters (a_{11}, a_{12}):

Case 1. For $a_{11} = -1$ and $a_{12} = 1$, the feasible steady states[1] of Eq. (S4) are

$$\mathcal{X} = \{(10, 0), (10.9761, 4.0835), (0, 5)\},$$

see Supplementary Figure 2a. In order to infer $\mathsf{sign}(\boldsymbol{J}_1)$, we focus on the line $L_{\{1\},\{1,2\}}$ connecting the samples $\boldsymbol{x}^{\{1\}} = (10, 0)$ and $\boldsymbol{x}^{\{1,2\}} = (10.9761, 4.0835)$ where taxon 1 is present. The line orthogonal to $L_{\{1\},\{1,2\}}$ (shown in green) determines the possible sign-patterns for $\boldsymbol{J}_1$. The sign-patterns corresponding to this orthogonal line are $\hat{\mathcal{S}}_1 = \{(-,+), (0,0), (+,-)\}$ and notice that $\mathsf{sign}(\boldsymbol{J}_1) \in \hat{\mathcal{S}}_1$.

Case 2. For $a_{11} = -1$ and $a_{12} = -1$, the feasible steady states of Eq. (S4) are

$$\mathcal{X} = \{(10, 0), (9.02381, 4.09976), (0, 5)\},$$

see Supplementary Figure 2b. We focus on the line orthogonal to $L_{\{1\},\{1,2\}}$ (shown in green) and obtain its corresponding sign-patterns $\hat{\mathcal{S}}_1 = \{(-,-), (0,0), (+,+)\}$. Again, notice that $\mathsf{sign}(\boldsymbol{J}_1) \in \hat{\mathcal{S}}_1$.

In the general case of N taxa we have the following result:

Theorem 2. Let $\mathcal{S}_{I,K} \subseteq \{-, 0, +\}^N$ be the set of all sign-patterns associated with the vectors orthogonal to $(\boldsymbol{x}^I - \boldsymbol{x}^K)$ and define $\hat{\mathcal{S}}_i = \bigcap_{\boldsymbol{x}^I,\boldsymbol{x}^K \in \mathcal{X}_i} \mathcal{S}_{I,K}$. Then $\mathsf{sign}(\boldsymbol{J}_i) \in \hat{\mathcal{S}}_i$.

Proof. From Assumption 2, we know that $\mathsf{sign}(\boldsymbol{J}_i) = \mathsf{sign}(\int_{I,K} \boldsymbol{J}_i)$ for all $\boldsymbol{x}^I, \boldsymbol{x}^K \in \mathcal{X}_i$. Then Eq. (S3) implies that $\mathsf{sign}(\boldsymbol{J}_i) \in \mathcal{S}_{I,K}$ for all pairs $\boldsymbol{x}^I, \boldsymbol{x}^K \in \mathcal{X}_i$. Thus, $\mathsf{sign}(\boldsymbol{J}_i)$ must belong to the intersection of all $\mathcal{S}_{I,K}$, implying that $\mathsf{sign}(\boldsymbol{J}_i)$ belongs to the sign-patterns shared by all $\mathcal{S}_{I,K}$. □

Remark 5.

[1]We say a steady state is feasible if it belongs to the orthant $\mathbb{R}^N_{\geq 0}$, that is, if no taxon has negative abundance.

a. In Theorem 2, in order to check if there is a vector with sign-pattern $\boldsymbol{s} \in \{-, 0, +\}^N$ orthogonal to a given vector $(\boldsymbol{x}^I - \boldsymbol{x}^K)$, we can check if the following linear program has a solution:

$$\text{Find } \boldsymbol{v} \in \mathbb{R}^N \text{ subject to } \boldsymbol{v}^\intercal(\boldsymbol{x}^I - \boldsymbol{x}^K) = 0 \text{ and } \mathsf{sign}(\boldsymbol{v}) = \boldsymbol{s}. \tag{S5}$$

Note that the condition $\mathsf{sign}(\boldsymbol{v}) = \boldsymbol{s}$ can be encoded as a set of equalities/inequalities of the form $\{v_i = 0,\ v_i < 0, v_i > 0\}$ corresponding to the cases $\{s_i = 0, s_i = -1, s_i = 1\}$. In Supplementary Note , we will show that it is possible to solve this problem more efficiently using the notion of sign-satisfaction.

b. From a geometrical viewpoint, the vectors $\boldsymbol{v} \in \mathbb{R}^N$ satisfying $\boldsymbol{v}^\intercal(\boldsymbol{x}^I - \boldsymbol{x}^K) = 0$ correspond to the hyperplane with normal vector $(\boldsymbol{x}^I - \boldsymbol{x}^K)$. Thus, the set of sign-patterns of those vectors $\boldsymbol{v}$ (i.e., the sign-patterns in the set $\mathcal{S}_{I,K}$) corresponds to the orthants of $\mathbb{R}^N$ crossed by this hyperplane. Consequently, $\hat{\mathcal{S}}_i = \bigcap_{\boldsymbol{x}^I, \boldsymbol{x}^K \in \mathcal{X}_i} \mathcal{S}_{I,K}$ corresponds to those orthants of $\mathbb{R}^N$ crossed by *all* hyperplanes orthogonal to $(\boldsymbol{x}^I - \boldsymbol{x}^K)$ for all $\boldsymbol{x}^I, \boldsymbol{x}^K \in \mathcal{X}_i$.

c. Note that $\mathbf{0} = (0, \cdots, 0) \in \hat{\mathcal{S}}_i$ always. Additionally, there is always at least three admissible sign-patterns in $\hat{\mathcal{S}}_i$, that is $\hat{\mathcal{S}}_i = \{-\hat{\boldsymbol{s}}_i, \mathbf{0}, \hat{\boldsymbol{s}}_i\}$ for some $\hat{\boldsymbol{s}}_i \in \{-, 0, +\}^N$.

d. A consequence of Remark 5.b above is that the steady-state data alone cannot be informative enough to determine a unique sign-pattern for $\boldsymbol{J}_i$. The number m of sign-patterns (candidate solutions) in $\hat{\mathcal{S}}_i$ depends on the number and informativeness of the samples $\{\boldsymbol{x}^I, I \in \mathcal{I}_i\}$ in the steady-state dataset $\mathcal{X}$. Indeed, if m sign-patterns are in $\hat{\mathcal{S}}_i$, only by providing the sign of exactly $\lfloor m/3 \rfloor$ non-zero entries of $\boldsymbol{J}_i$ as prior information, we can univocally infer $\mathsf{sign}(\boldsymbol{J}_i)$. For instance, in the case of $N = 2$ taxa in Example 2 where $m = 3$, we need to provide the sign of only one non-zero entry of $\boldsymbol{J}_i$, and that will let us infer the sign of the other entry.

To conclude this subsection, we discuss some implications of Assumption 2 on the steady-state samples $\mathcal{X}$ that can be observed in the microbial community. Let $\mathcal{E} := \{x \in \mathbb{R}^N | x_i f_i(x) = 0; i = 1, \cdots, N\} \subseteq \mathbb{R}^N$ denote the set of equilibria of Eq. (S1).

Definition 1. System (S1) is said to have *true multi-stability* if $\mathcal{E}$ contains at least two isolated sets (i.e., two sets that don't intersect) of interior equilibria.

A particular case of true multi-stability is when the system or any subsystem (composed of a particular subset of taxa) exhibits multiple interior equilibria, where all the involved taxa have positive abundances.

Proposition 1. If Assumption 2 is satisfied then there is no true multi-stability.

Proof. We argue by contradiction. Suppose that Assumption 2 is satisfied but the system has true multi-stability. Let $\boldsymbol{x}^I$ and $\boldsymbol{x}^K$ be two interior equilibria belonging to two different isolated sets in $\mathcal{E}$. Suppose they are interior with respect to the i-th taxon. Now consider the scalar function $p(\sigma) = f_i(\boldsymbol{x}^I + \sigma(\boldsymbol{x}^K - \boldsymbol{x}^I))$. Note that $p(\sigma)$ is a non-zero meromorphic function because $\boldsymbol{x}^I$ and $\boldsymbol{x}^K$ belong to different isolated sets of equilibria. Furthermore, $p(0) = p(1) = 0$ as both $\boldsymbol{x}^I$ and $\boldsymbol{x}^K$ are interior equilibria for $f_i(x)$. Together, this implies that the slope of $p(\sigma)$ needs to change sign at least once. Since the slope of $p(\sigma)$ equals the Jacobian of f_i in the direction of the vector $\boldsymbol{x}^I - \boldsymbol{x}^K$, this implies that the Jacobian changes sign, contradicting Assumption 2. □

Remark 6.

a. Proposition 1 actually provides a simple criterion to falsify Assumption 2. Namely, if a microbial community displays true multi-stability, then Assumption 2 is invalid, i.e., the sign-pattern of its Jacobian matrix is not constant. In practice, we can detect the presence of true multi-stability in the available samples $\mathcal{X}$ (e.g., two or more steady-state samples have the same collection of present taxa but totally different abundance profiles). If multi-stability is detected, then we know immediately that Assumption 2 is invalid. If multi-stability is not detected, then at least Assumption 2 is consistent with the collected steady-state samples.

In short, by introducing a criterion to falsify our assumption, we significantly enhance the applicability of our method for inferring interaction types.

b. In case the sign-pattern of the Jacobian matrix is not constant but the steady-state samples were still collected from a microbial community under the same or similar environmental conditions (e.g., nutrient availability), we can interpret our inferred sign-patterns as the overall inhibition or promotion effect between different taxa during the transitions between steady states. To see this point, note that regardless of the sign-pattern of the Jacobian matrix is constant or not, our method can correctly infer the sign of $\int_0^1 J_{ij}(\boldsymbol{x}^I + \sigma(\boldsymbol{x}^K - \boldsymbol{x}^I))d\sigma$, which reflects an overall impact (inhibition or promotion) of taxon j on taxon i during the transition from the steady state $\boldsymbol{x}^I$ $(\sigma = 0)$ to $\boldsymbol{x}^K$ $(\sigma = 1)$.

Example 4. To illustrate Remark 6.b, let's consider a toy model of two species X and Y. Each species has a per capita growth rate that is modulated by its mutualistic partner as well as the resource. The population dynamics of this toy model is given by

$$\begin{cases} \dot{X} = r_x X(\frac{Y+a}{Y+a+\kappa})(1-X-Y) - \delta X, \\ \dot{Y} = r_y Y(\frac{\beta X+a}{\beta X+a+\kappa})(1-X-Y) - \delta Y. \end{cases} \tag{S6}$$

Here r_x and r_y are the growth rates of the species, a is the amount of resource, δ is the death rate, κ is an effective Monod constant, and $\beta > 0$ quantifies the asymmetry of benefit that each species receives from its partner. The elements of the Jacobian matrix of this community are given by

$$\begin{cases} J_{11} = -r_x \frac{Y+a}{Y+a+\kappa}, \\ J_{12} = r_x(1-X-Y)\frac{\kappa}{(Y+a+\kappa)^2} - r_x\frac{Y+a}{Y+a+\kappa}, \\ J_{21} = r_y(1-X-Y)\frac{\beta\kappa}{(\beta X+a+\kappa)^2} - r_y\frac{\beta X+a}{\beta X+a+\kappa}, \\ J_{22} = -r_y\frac{\beta X+a}{\beta X+a+\kappa}. \end{cases}$$

Note that J_{11} and J_{22} are always negative, while J_{12} and J_{21} may change their signs depending on the particular abundances of X and Y, as well as the model parameters. This model captures the transition between the different regimes of ecological interaction depending on the amount of resource (determined by a). Indeed, Supplementary Figure 3a shows that there are three regimes with different inter-species interactions starting from mutualism and then leading to parasitism and competition. Here, the overall or "effective" interaction types are determined by comparing the difference of steady states between monocultures (dashed lines in Supplementary Figure 3a) and co-cultures (solid lines in Supplementary Figure 3a). This allow us to calculate the relative yield, which indicates the promotion or inhibition impact between two taxa. For this model, because that Jacobian may change its sign-pattern over time, the sign of relative yields can be interpreted as the effective impact between two taxa, denoted as J_{eff} (Supplementary Figure 3a).

We now apply our method using steady states of this model. Supplementary Figure 3b-d shows the diagrams of our inference method under different resource amounts. We found that the inferred inter-species interaction types are consistent with the ground truth (shown in Supplementary Figure 3a). Supplementary Figure 3e-g shows the value of $J_{12}(\boldsymbol{x}^{\{1,2\}} + \sigma(\boldsymbol{x}^{\{2\}} - \boldsymbol{x}^{\{1,2\}}))d\sigma$ and $J_{21}(\boldsymbol{x}^{\{1,2\}} + \sigma(\boldsymbol{x}^{\{2\}} - \boldsymbol{x}^{\{1,2\}}))d\sigma$ as a function of σ corresponding to the transition between $\boldsymbol{x}^{\{1,2\}}$ $(\sigma = 0)$ and $\boldsymbol{x}^{\{2\}}$ $(\sigma = 1)$. The shade areas in this figure denote the value of $\int_0^1 J_{12}(\boldsymbol{x}^{\{1,2\}} + \sigma(\boldsymbol{x}^{\{2\}} - \boldsymbol{x}^{\{1,2\}}))d\sigma$ and $\int_0^1 J_{21}(\boldsymbol{x}^{\{1,2\}} + \sigma(\boldsymbol{x}^{\{2\}} - \boldsymbol{x}^{\{1,2\}}))d\sigma$. For example, when $a = 0.15$, both $\int_0^1 J_{12}(\boldsymbol{x}^{\{1,2\}} + \sigma(\boldsymbol{x}^{\{2\}} - \boldsymbol{x}^{\{1,2\}}))d\sigma$ and $\int_0^1 J_{21}(\boldsymbol{x}^{\{1,2\}} + \sigma(\boldsymbol{x}^{\{2\}} - \boldsymbol{x}^{\{1,2\}}))d\sigma$ are positive (shaded areas in Supplementary Figure 3e). Although J_{12} and J_{21} display both negative and positive values as σ changes, the positive J_{12} and J_{21} dominate in the transition between two steady states. Hence, overall taxon X and taxon Y

are mutualistic. Supplementary Figure 3f,g shows the result of this analysis applied to J_{12} and J_{21} for $a = 0.2$ and $a = 0.5$.

Supplementary Note 2: Brute-force and heuristic algorithms

Here we introduce the methodology for inferring the zero- or sign-patterns of the Jacobian matrix associated with the population dynamics of a microbial community. In essence, the inference of the zero-pattern is similar to the inference of the sign-pattern. Indeed, the only difference is that the former doesn't care if the non-zero values are positive or negative. This implies that the complexity for inferring the network topology and interaction types are roughly the same. Here, for simplicity we describe the algorithms for inferring sign-patterns. All the algorithms (and pseudo codes) can be easily modified to infer the zero-pattern.

Brute-force algorithm. Theorems 1 and 2, together with Remarks 3 and 5 can be used to construct an algorithm to obtain all admissible sign-patterns for given steady-state data. Indeed, by enumerating all possible sign-patterns, we can use the liner program in Eq. (S5) to check if each of the possible 3^N sign-patterns is admissible for taxon i, see Algorithm 1.

Algorithm 1 A brute-force algorithm to compute $\hat{\mathcal{S}}_i$

Input:

The collection of matrices M_i, being the difference between all two samples containing species i. $M_i \in \mathbb{R}^{\binom{N\times|\mathcal{I}_i|}{2}}$, $|\mathcal{I}_i|$ is the number of samples

Output:

The sign-pattern set of $\hat{\mathcal{S}}_i$

```
1: Ê_i ← Enumeration of all the possible combinations of {−, 0, +}^N
2: for each j-th row in M_i do
3:    S_j ← ∅
4:    for each kth-subset in Ê_i do
5:       if find v ∈ ℝ^N subject to v^T M_i[j, :] = 0 and sign(v) = Ê_i[k] then
6:          S_j = S_j ⋃ Ê_i[k]
7:       end if
8:    end for
9:    Ŝ_i = ⋂_j S_j
10: end for
```

Below, we illustrate the application of the brute-force algorithm for a microbial community with $N = 3$ taxa.

Example 5. Here we consider the case of a microbial community with $N = 3$ taxa and population dynamics given by the so-called Crowley-Martin functional response [5]. The ODEs are:

(S7)
$$\begin{cases} \dot{x}_1 = x_1\left(r_1 + a_{11}x_1 + a_{12}\dfrac{x_2}{0.1h + 9x_1 + 6x_1x_2 + x_2} + a_{13}\dfrac{x_3}{2h + 0.1x_1 + 0.4x_1x_3 + x_3}\right), \\ \dot{x}_2 = x_2\left(r_2 + a_{21}\dfrac{x_1}{h + x_1 + 2.5x_1x_2 + 2x_2} + a_{22}x_2 + a_{23}\dfrac{x_3}{0.5h + 2.3x_2 + 1.1x_2x_3 + x_3}\right), \\ \dot{x}_3 = x_3\left(r_3 + a_{31}\dfrac{x_1}{0.5h + x_1 + 10x_1x_3 + 4x_3} + a_{32}\dfrac{x_2}{2.1h + x_2 + 0.2x_2x_3 + 0.5x_3} + a_{33}x_3\right). \end{cases}$$

We set the parameters $r_1 = 1, r_2 = 5, r_3 = 1.5$, $h = 0.2$ and

$$A = \begin{pmatrix} -1 & 0 & 3 \\ 0 & -1 & 0 \\ 0.5 & 5 & -0.4 \end{pmatrix}.$$

Notice again that $\mathsf{sign}(J) = \mathsf{sign}(A)$. We focus on reconstructing $\mathsf{sign}(\boldsymbol{J}_1)$, as the same procedure applies to the other taxa. With the given parameters, the feasible steady states of Eq. (S7) where

taxon 1 is present are:

$$\mathcal{X}_1 = \{(1., 0., 0.), (2.4069, 0., 3.77772), (2.45168, 5., 7.51002), (1., 5., 0)\},$$

constituting the available steady-state samples for taxon 1. We apply Algorithm 1 to this dataset obtaining

$$\hat{\mathcal{S}}_1 = \{(-, 0, +), (0, 0, 0), (+, 0, -)\}.$$

Note that $\mathsf{sign}(\boldsymbol{J}_1) \in \hat{\mathcal{S}}_1$. Providing for example $\mathsf{sign}(J_{11}) < 0$ as prior information, we correctly infer that $\mathsf{sign}(J_1) = (-, 0, +)$.

Inference using the heuristic algorithm. In practice, for large microbial communities with unknown dynamics, the inference of ecological interactions according to Algorithm 1 has two major drawbacks: (i) Checking if an orthant is crossed by a given hyperplane using the linear program of Eq. (S5) is computationally expensive. (ii) The number of orthants that is necessary to check (i.e., the solution space) increases as 3^N, that is, exponentially in the number of taxa.

To circumvent the first drawback, we introduce an alternative method based on the notion of sign-satisfaction. To address the second challenge, we propose a heuristic algorithm with user-defined time complexity to infer the sign-pattern of $\boldsymbol{J}_i$.

Formulating the sign-satisfaction problem. Consider a real-valued vector $\boldsymbol{y} \in \mathbb{R}^N$. Then, solving the linear program Eq. (S5) is equivalent to solving the following sign-satisfaction problem:

$$\text{(S8)} \qquad \text{Find } \mathsf{sign}(\boldsymbol{y}) \in \{-, 0, +\}^N \text{ subject to } \boldsymbol{y}^\intercal(\boldsymbol{x}^I - \boldsymbol{x}^K) = 0.$$

Notice that from a geometrical viewpoint, solving Eq. (S8) is just finding the orthants of $\mathbb{R}^N$ crossed by the hyperplane orthogonal to $(\boldsymbol{x}^I - \boldsymbol{x}^K)$.

In the next example, we illustrate how the sign-satisfaction formulation allows us to quickly discard orthants of $\mathbb{R}^N$ that cannot be crossed by such hyperplane:

Example 6. In vector form, Eq. (S3) in Example 5 can be written as

$$\left(\int_{\{1\},\{1,3\}} J_{11} d\sigma, \ \int_{\{1\},\{1,3\}} J_{12} d\sigma, \ \int_{\{1\},\{1,3\}} J_{13} d\sigma\right)^\intercal \cdot (-1.4069, \ \ 0, \ \ -3.77772) = 0$$

if we take two samples sharing taxon 1. Thus, the sign-satisfaction for Example 5 can be written as

$$\text{Find } \mathsf{sign}(\boldsymbol{y}) \in \{-, 0, +\}^3 \text{ subject to } \boldsymbol{y}^\intercal(-1.4069, \ \ 0, \ \ -3.77772) = 0.$$

Note that, for example, the choice $\mathsf{sign}(\boldsymbol{y}) = (-, 0, -)$ cannot satisfy the above condition regardless of the particular value of $\boldsymbol{y}$, because the inner product is the sum of two positive numbers, which can never be zero.

A systematic method to extend the above example and solve the sign-satisfaction problem is discussed next.

A graph-based approach to solving the sign-satisfaction problem. We illustrate the basic idea using a small example, and then discuss the general case.

In Example 6, the sign-satisfaction problem required that

$$(-1.4069 \times y_1) + (0 \times y_2) + (-3.77772 \times y_3) = 0,$$

where $\mathsf{sign}(y_1, y_2, y_3) = \mathsf{sign}(J_{11}, J_{12}, J_{13})$. We map the above equation to the sign-satisfaction graph in Supplementary Figure 4, where each element of $\boldsymbol{J}_1$ corresponds to a column and each element of $\mathsf{sign}(\boldsymbol{J}_1)$ has three possibilities (i.e., '−','0' or '+'). Each node in Supplementary Figure 4 is divided in two parts: the left is an entry of $\mathsf{sign}(\boldsymbol{x}^I - \boldsymbol{x}^K)$ and the right is an entry of $\mathsf{sign}(\boldsymbol{J}_1)$. The color of each node encodes the sign of the product of left and right parts: grey is zero, red is positive and blue is negative. Next we introduce edges starting from each node and pointing to all nodes located

in the next column to its right. With this formulation, the solutions to the sign-satisfaction problem (S8) reduces to finding the paths in the sign-satisfaction graph that satisfy one of the following two conditions: (i) the path contains red (representing positive values) and blue (representing negative values) nodes simultaneously, or(ii) the path contains only gray (representing zero values) nodes.

The above two conditions guarantee that the sum of the product of $\mathsf{sign}(J_{ij})$ and $(\boldsymbol{x}^I - \boldsymbol{x}^K)$ can be zero. At this step, it is also useful to introduce the prior information that is available, such as $J_{ii} < 0$, allowing us to collapse columns of nodes in the sign-satisfaction graph to single nodes (Supplementary Figure 4).

In a general case, for a given $(\boldsymbol{x}^I - \boldsymbol{x}^K)$, the construction of the sign-satisfaction graph is as follows: (i) the graph consists of N columns, with each column having three nodes; (ii) each node in the graph is divided into two parts: the left correspond to an entry of $\mathsf{sign}(\boldsymbol{x}^I - \boldsymbol{x}^K)$ and the right to an entry of $\mathsf{sign}(\boldsymbol{J}_i)$; (iii) each node is colored according to the sign of the product of the left and right parts: zero is grey, positive is red and negative is blue; (iv) directed edges are included from a node to all the nodes in next column.

Finally, a solution to the sign-satisfaction problem of Eq. (S8) corresponds to a path from the first column to N-th column satisfying either condition (i) or (ii) listed above. In such case, a possible sign-pattern of $\boldsymbol{J}_1$ consists of the sign in the right part of each node in the path. For instance, the paths with yellow directed edges in Supplementary Figure 4 correspond to the possible sign-pattern of $\boldsymbol{J}_1$, i.e., $(-, +, +)$, $(-, -, +)$ and $(-, 0, +)$.

By using the sign-satisfaction graph, it is very efficient to test if the hyperplane orthogonal to $(\boldsymbol{x}^I - \boldsymbol{x}^K)$ crosses some orthants of $\mathbb{R}^N$, because it reduces to checking if its corresponding vector in $\{-, 0, +\}^N$ satisfies either condition (i) or (ii). However, finding all orthants crossed by such orthogonal hyperplane remains challenging, since the sign-satisfaction graph did not decrease the dimension of the solution space (that remains with exponential size 3^N). To address this issue, next we introduce a method to efficiently sample paths in the sign-satisfaction graph.

Use the intersection of hyperplanes to sample paths in the sign-satisfaction graph. As discussed before, with the sign-satisfaction graph the solution space is still exponential (with size 3^{N-1}, where the term $N - 1$ comes from assuming we know that $J_{ii} < 0$ as prior information). One possibility to circumvent this problem would be to randomly sample paths in the sign-satisfaction graph and check if they satisfy conditions (i) or (ii). This would not work, however, since the probability of sampling the true "$\mathsf{sign}(\boldsymbol{J}_i)$" is only $X/3^{N-1}$ — where X is the number of sampled paths — and this probability approaches zero as N increases. To alleviate this problem, next we propose a method to sample paths in the sign-satisfaction graph with certain preference.

This method has the following four steps:

(step-1) **Construct the matrix of the difference of all the sample pairs.** Consider the set of all vectors $\{\boldsymbol{x}^I - \boldsymbol{x}^K | \boldsymbol{x}^I, \boldsymbol{x}^K \in \mathcal{X}_i\}$. Let $M_i \in \mathbb{R}^{N \times \binom{|\mathcal{X}_i|}{2}}$ be a matrix constructed by stacking all the $\binom{|\mathcal{X}_i|}{2}$ vectors, where $|\mathcal{X}_i|$ is the number of samples containing taxon i. By construction, each column of M_i is the normal vector of a hyperplane orthogonal to the difference of the corresponding sample pair.

(step-2) **Randomly sample $(N - 1)$ hyperplanes.** Choose randomly $N - 1$ columns from M_i.

(step-3) **Find the intersection of the $(N - 1)$ sampled hyperplanes to obtain an intersection line.** This can be done by finding the kernel of the matrix obtained by stacking the chosen columns. Note that the randomly sampled $(N - 1)$ hyperplanes not always intersect in a line, because some hyperplanes might be parallel. However, this situation is non-generic in $\mathbb{R}^N$. Thus, if the randomly sampled hyperplanes do no intersect as a line, we return to step 2 and choose a new subset of columns.

(step-4) **Count how many hyperplanes cross the region of the intersection line using the sign-satisfaction graph.** The sign-pattern of this intersection line represent the three orthants in $\mathbb{R}^N$ crossed by all those $(N - 1)$ hyperplanes. For the remaining hyperplanes in M_i (i.e., the rest of the columns in M_i), let $\tilde{\phi}$ be the number of those hyperplanes that cross these three orthants. We normalize $\tilde{\phi}$ using $\phi = \tilde{\phi}/\binom{|\mathcal{X}_i|}{2}$, so that $\phi \in [0, 1]$. Notice that $\phi = 1$ means that this sign-pattern of the

intersection line meets the requirements of sign-satisfaction for all the sample pairs. Therefore, the magnitude of the computed ϕ can be seen as the confidence of this potential solution to be a solution of the sign-satisfaction problem.

(step-5) **Go back to step-2 until $\Psi \geq 1$ intersection lines have been computed**.

In summary, selecting the intersection line can be seen as a "preference" sampling in the sign-satisfaction graph, because this intersection line can be crossed by at least $(N-1)$ hyperplanes in M_i.

We illustrate the basic idea of the above discussion in the following example:

Example 7. We compute the difference vector of all the sample pairs (the samples contains taxon 1) in Example 5 and stack them in the following matrix:

$$M_1 = \begin{pmatrix} -1.4069 & -1.4517 & 0 & -0.0448 & 1.4069 & 1.4517 \\ 0 & -5.0000 & -5.0000 & -5.0000 & -5.0000 & 0 \\ -3.7777 & -7.5100 & 0 & -3.7323 & 3.7777 & 7.5100 \end{pmatrix}.$$

Each column of M_1 is the difference of a sample pair, corresponding to the normal vector of a plane orthogonal to the associated $(\boldsymbol{x}^I - \boldsymbol{x}^K)$. In Supplementary Figure 5a, the intersection line (black line) is intersected by the planes where each of normal vectors respectively corresponds to the 1-st and 5-th column of the above M_1. The black line crosses the regions with sign-pattern $(-,0,+)^\intercal$ and $(+,0,-)^\intercal$. At least these two regions have been crossed by two planes. Due to the fact that we know that $J_{11} < 0$, for the next step we need to count the number of the remaining hyperplanes that cross the region with the sign-pattern $(-,0,+)^\intercal$. In Supplementary Figure 5b we find that four of the remaining hyperplanes cross this intersection line, that is, the normalized ϕ satisfies $\phi = 1$. It means that the sign-pattern of this intersection line is the inference of $\mathsf{sign}(\boldsymbol{J}_1)$ because it meets the requirements of sign-satisfaction for all the sample pair.

The heuristic algorithm combing sign-satisfaction and intersection of hyperplanes. Combining the sign-satisfaction graph with the sampling procedure described above, we propose a heuristic algorithm to infer the sign-pattern of $\boldsymbol{J}_i$.

Our heuristic algorithm has two inputs: the steady-state dataset for the i-th taxon $\mathcal{X}_i$ and a user-defined parameter Ψ determining how many intersection lines of hyperplanes will be constructed. The algorithm has of four steps, as described in Supplementary Figure 6. Applying this procedure for $i = 1, \ldots, N$, we can get the sign-pattern of the whole Jacobian matrix.

In summary, the algorithm works as follows. After generating an intersection line, we get the three orthants corresponding to this intersection line. Then we count how many hyperplanes cross the orthants determined by this intersection line using the sign-satisfaction graph, and this count can be normalized as $\phi \in [0,1]$ indicating the confidence of this potential solution to be a solution of final inference. Finally, we select the intersection line with the maximal ϕ among the generated Ψ intersection lines as the final inferred sign-pattern $\hat{\mathcal{S}}_i$.

Note that if the algorithm is stuck in generating an intersection line for some subset of $(N-1)$ hyperplanes, the heuristic algorithm will fail. Numerical experiments suggest this situation happens only when the data is not informative enough or the number of samples is smaller than the threshold Ω^*. In Fig. 3 of the main text, we presents the results of the minimal number of samples Ω^* required for a community with size N.

Supplementary Note 3: Inferring the topology of ecological networks

In essence, inferring the zero-pattern is similar to inferring the sign-pattern. Indeed, it is only necessary to recognize any non-zero entry of the inferred sign-pattern as a non-zero entry in the inferred zero-pattern. Notice how the zero-pattern corresponds to hyperplanes exactly aligned to the orthants of $\mathbb{R}^N$. Therefore, any measurement noise will make the difference of sample pairs deviate from the axis, easily leading to inference errors (see Supplementary Figure 1d,f). To alleviate this problem, we introduce a user-defined cutoff value to judge the zero-pattern of J_{ij} based on the angle between the axis and the intersection line (Supplementary Figure 1).

For the brute-force method, first we set an element in the difference of sample pair $\boldsymbol{x}^I - \boldsymbol{x}^K$ to 0 if the magnitude of that element is less than the user-defined cutoff. Second, we construct the hyperplanes respectively orthogonal to these modified difference of sample pairs. Third, we count how many hyperplanes cross each orthant in the $\mathbb{R}^N$. Finally, we select the region crossed by the maximal hyperplanes as the inferred zero-pattern. Recall that the brute-force method is limited to infer the microbial community with $N \leq 10$.

For larger microbial communities, we also developed a heuristic algorithm that is very similar to our heuristic algorithm for inferring the sign-pattern. In that algorithm, notice how the deviation of an intersection line from an axis is directly given by its directional vector. Indeed, this vector contains the cosine of the angles between the axis and the intersection line. This algorithm works as follows:

(step-1) **Construct the matrix of the difference of all the sample pairs.** Consider the set of all vectors $\{\boldsymbol{x}^I - \boldsymbol{x}^K | \boldsymbol{x}^I, \boldsymbol{x}^K \in \mathcal{X}_i\}$. Let $M_i \in \mathbb{R}^{N \times \binom{|\mathcal{X}_i|}{2}}$ be a matrix constructed by stacking all the $\binom{|\mathcal{X}_i|}{2}$ vectors, where $|\mathcal{X}_i|$ is the number of samples containing taxon i. By construction, each column of M_i is the normal vector of a hyperplane orthogonal to the difference of the corresponding sample pair.

(step-2) **Randomly sample $(N-1)$ hyperplanes.** Choose randomly $N-1$ columns from M_i.

(step-3) **Find the intersection of the $(N-1)$ sampled hyperplanes to obtain an intersection line.** This can be done by finding the kernel of the matrix obtained by stacking the chosen columns. Note that the randomly sampled $(N-1)$ hyperplanes not always intersect in a line, because some hyperplanes might be parallel. However, this situation is non-generic in $\mathbb{R}^N$. Thus, if the randomly sampled hyperplanes do no intersect as a line, we return to step 2 and choose a new subset of columns.

(step-4) **Set the elements in the directional vector of this intersection line as zero if their absolute values are less than the cutoff value. Then we get a new directional vector.** Note that we scale the 2-norm of this directional vector to 1. The absolute value of i-th element in the directional vector represents the cosine of the angle between the intersection line and x_i-axis. If the value is large enough, it means the intersection line almost locates at the x_i-axis. Therefore, if the absolute value of directional vector is smaller than the cutoff, we set this entry to 0.

(step-5) **Count how many hyperplanes cross the region of the new directional vector using the sign-satisfaction graph.** The sign-pattern of new directional vector represent the orthants in $\mathbb{R}^N$ crossed by all those $(N-1)$ hyperplanes. For the rest hyperplanes of M_i (i.e., the rest of the columns in M_i), let $\tilde{\phi}$ be the number of those hyperplanes that cross the orthants. We normalize $\tilde{\phi}$ using $\phi = \tilde{\phi}/\binom{|\mathcal{X}_i|}{2}$, so that $\phi \in [0, 1]$. Notice that $\phi = 1$ means that the sign-pattern of the new directional vector meets the requirements of sign-satisfaction for all the sample pairs. Therefore, the magnitude of the computed ϕ can be seen as the confidence of this potential solution to be a solution of the sign-satisfaction problem.

(step-6) **Go back to step-2 until $\Psi \geq 1$ intersection lines have been computed**.

We validated this method using steady-state data generated from four different population dynamics. Except the Generalized Lotka-Volterra (GLV), the other three population dynamics models have non-linear functional responses: Holling Type II (H), DeAngelis-Beddington (DB) and Crowley-Martin (CM). Supplementary Figure 7 shows the inferred network topology on four different population dynamics models. We found that in case the noise level is $\eta = 0.1$, the accuracy of inference

can be around 0.8, if the cutoff is between 0.1 and 0.2. However, in the noiseless case, increasing the cutoff can decrease the accuracy, because larger cutoff induces more false positives of interactions.

Supplementary Note 4: Inferring the ecological interaction types

Using the brute-force method to infer the interaction types is deterministic because we search all the combinations of $\{+, 0, -\}^N$, and accuracy increases with the increment of sample size. However, due to the time complexity, application of the brute-force method is limited to small microbial communities, e.g., $N \leq 10$. This motivated us to develop the heuristic method of Supplementary Note that is suitable for larger microbial communities.

To validate the effectiveness of our heuristic algorithm, we tested it using simulated steady-state data generated by models of the form Eq. (S1). In particular, we considered a model with pair-wise interactions of the form

$$\dot{x}_i = x_i \left[r_i + \sum_{j=1}^{N} a_{ij} g(x_i, x_j) \right], \quad i = 1, \cdots, N, \tag{S9}$$

where $r_i \in \mathbb{R}$ is the intrinsic growth rate of the i-th taxon, $A = (a_{ij}) \in \mathbb{R}^{N \times N}$ is a constant matrix and the function $g(x_i, x_j) : \mathbb{R} \times \mathbb{R} \to \mathbb{R}$ is the so-called *functional response* [1, 2, 3, 4, 5, 6]. Recall that these functional responses model the intake rate of a consumer as a function of food density, and thus different functional responses correspond to different mechanisms of interaction between taxa.

We used Eq. (S9) to generate synthetic steady-state datasets for 4 different functional responses with different complexity. The first was the linear functional response

$$g_{\text{LV}}(x_i, x_j) = x_j,$$

for which Eq. (S9) actually reduces to the classical Generalized Lotka-Volterra (GLV) model. In this case, the accuracy of the heuristic algorithm on inferring the sign-pattern $\mathsf{sign}(J) = \mathsf{sign}(A)$ is 100% if there are enough steady-state samples, see Fig. 3a in the main text. Indeed, this is a consequence of the following proposition:

Proposition 2. In the noiseless case, if the functional response is linear, the directional vector of intersection line of any $(N-1)$ hyperplanes orthogonal to $\{\boldsymbol{x}^I - \boldsymbol{x}^K | I, K \in \mathcal{I}_i\}$ is the same and parallel to $\boldsymbol{J}_i$.

Proof. Due to the fact that the functional response is linear, the Jacobian matrix become simple and constant for different samples, that is, $J = A$. Therefore, Eq. (S3) is equal to

$$\boldsymbol{a}_i M_i = \boldsymbol{0},$$

where $M_i \in \mathbb{R}^{N \times \binom{|\mathcal{I}_i|}{2}} = \{\boldsymbol{x}^I - \boldsymbol{x}^K | \forall (I, K) \in \mathcal{I}_i\}$ denotes the difference of all sample pairs. As we know, $\boldsymbol{a}_i \neq \boldsymbol{0}$, representing the interaction vector in the A matrix, is unique to M_i. Thus the non-trivial solution of $\boldsymbol{a}_i$ in the above equation array must meet the requirement

$$\text{rank}(M_i) = N - 1.$$

That is to say, if we randomly select $(N-1)$ columns in M_i as $\bar{M}_i \in \mathbb{R}^{N \times (N-1)}$, then

$$\boldsymbol{a}_i \bar{M}_i = \boldsymbol{0}.$$

Actually, the randomly selected $\bar{M}_i$ corresponds to $(N-1)$ hyperplanes respectively orthogonal to each columns of $\bar{M}_i$ in the geometric perspective. The directional vector of intersection line of these $(N-1)$ hyperplanes can be calculated by $\text{null}(\bar{M}_i^\top) \in \mathbb{R}^N$, which is parallel to $\boldsymbol{a}_i$. □

The remaining three functional response were Holling Type II (H), DeAngelis-Beddington (DB) and Crowley-Martin (CM), given by the following equations

$$g_{\text{H}}(x_i, x_j) = \frac{c_1 x_j}{1 + c_1 c_2 x_j}, \quad g_{\text{DB}}(x_i, x_j) = \frac{c_1 x_j}{1 + c_1 c_2 x_i + c_3 x_j}, \quad g_{\text{CM}}(x_i, x_j) = \frac{c_1 x_j}{(1 + c_1 c_2 x_i)(1 + c_3 x_j)}.$$

Here c_1, c_2, c_3 are constants. Note that these nonlinear functional responses lead to more complicated population dynamics. For the results presented in Fig. 3 of the main text, we used $c_1 = 1, c_2 = c_3 = 0.1$. Those results show that the heuristic algorithm accurately infers the sign-pattern of Jacobian matrix for these three functional responses and its accuracy is above 95%.

Supplementary Note 5: Inferring interaction strengths with GLV dynamics

A particular class of systems in (S1) is when the Jacobian J_i is constant, implying that $f_i(x) = \boldsymbol{a}_i^\intercal \boldsymbol{x} + r_i$ for some constant vector $\boldsymbol{a}_i \in \mathbb{R}^N$ and scalar r_i. In such case, the system reduces to the Generalized Lotka-Volterra (GLV) model

$$\dot{x}_i = x_i\,(\boldsymbol{a}_i^\intercal \boldsymbol{x} + r_i), \quad i = 1, \cdots, N, \tag{S10}$$

where $\boldsymbol{a}_i = (a_{ij}) \in \mathbb{R}^N$ is the i-th row of the so-called interaction matrix $A \in \mathbb{R}^{N\times N}$, and $r_i \in \mathbb{R}$ is the intrinsic growth rate of taxon i. As discussed in the main text, the GLV models also allows defining the *interaction strength* of taxon j on taxon i as a_{ij}.

A condition for detecting GLV dynamics. Our first observation is that the steady-state samples $\mathcal{X}$ can be used to decide if they could be produced by a GLV model:

Theorem 3. A necessary condition for the dynamics of the i-th species to be GLV is that all samples $\{\boldsymbol{x}^I \in \mathcal{X}, I \in \mathcal{I}_i\}$ align into a hyperplane.

Proof. If for all $I \in \mathcal{I}_i$ the samples x^I align into a hyperplane, then $f_i(x)$ should be a hyperplane whose general equation is $a_i^\intercal x + r_i$. □

As discussed in the main text, with real data containing measurement noises and other errors, the samples will not align exactly into a hyperplane. In such case, the coefficient of determination (denoted by R^2) of a hyperplane fitted to the samples containing taxon i can be used to judge if its dynamics can be adequately described by the GLV model. For a given dataset $\mathcal{X}$, if the average of R^2 of the hyperplanes fitted to the samples of the i-th taxon is > 0.9, then we consider that it is possible to infer the inter-taxa interaction strengths and intrinsic growth rates using the GLV model for this taxon. Otherwise, we recommend to infer only the interaction types. The pipeline for detecting GLV dynamics is described as Supplementary Figure 8.

Inference of interaction strengths and intrinsic growth rates. Under the GLV model, Eq. (S3) reduces to

$$\boldsymbol{a}_i^\intercal \cdot (\boldsymbol{x}^I - \boldsymbol{x}^K) = 0, \quad \forall (I, K) \in \mathcal{I}_i. \tag{S11}$$

If we denote by P_i the $(N-1)$ dimensional hyperplane spanned by all the steady-state samples sharing the i-th taxon $\mathcal{X}_i$, Eq. (S11) implies that the $\boldsymbol{a}_i$ belongs to the one-dimensional space orthogonal to P_i. Thus the normal vector of the fitted hyperplane according to $\mathcal{X}_i$ is parallel to $\boldsymbol{a}_i$. To infer the precise value of interaction strengths, additional prior information, at least one non-zero element in $\boldsymbol{a}_i$, is needed. Otherwise, we can only infer the relative strength of the interactions between taxa.

Applying the Knockoff filter to control the false discovery rate. Eq. (S11) shows that $\boldsymbol{a}_i$ can be inferred by fitting a hyperplane based on all the steady-state samples sharing the i-th taxon $\mathcal{X}_i = \text{row}\{\boldsymbol{x}^I \in \mathbb{R}^N, I \in \mathcal{I}_i\}$, provided that we know at least one non-zero element in $\boldsymbol{a}_i$, say a_{ii} (or an estimate $\hat{a}_{ii}$ of it). Consider that the ecological network to be inferred is sparse. Then, a natural method to find a sparse solution is by using the so-called Lasso regression:

$$\min_{\beta\in\mathbb{R}^N} \left\{ \frac{1}{N} \|\boldsymbol{y} - X\boldsymbol{\beta}\|_2^2 + \lambda \|\boldsymbol{\beta}\|_1 \right\},$$

where λ is the Lasso (regularization) parameter. Here $\boldsymbol{y}$ is the i-th column of $\mathcal{X}_i$, and

$$X = \text{col}\{\boldsymbol{x}_1, \cdots, \boldsymbol{x}_{i-1}, \boldsymbol{x}_{i+1}, \cdots, \boldsymbol{x}_N, \boldsymbol{1}, \} \in \mathbb{R}^{|\mathcal{I}_i|\times N}$$

is the matrix obtained from $\mathcal{X}_i$ by deleting the i-th column and adding $\boldsymbol{1}$ in the end. $\boldsymbol{x}_i$ is the i-th column of $\mathcal{X}_i$. This structure happens because for the GLV we have $r_i + a_{i1}x_1 + \cdots + a_{ii}x_i + a_{i,i+1}x_{i+1} + \cdots + a_{iN}x_N = 0$ and we assumed for the numerical results that $a_{ii} = -1$. Once a solution $\boldsymbol{\beta}$ to the above Lasso problem is found, the estimation $\hat{\boldsymbol{a}}_i$ for $\boldsymbol{a}_i$ is given by

$$\hat{\boldsymbol{a}}_i = \hat{a}_{ii}\,[\boldsymbol{\beta}(1:j-1), -1, \boldsymbol{\beta}(j:N-1)],$$

where $\boldsymbol{\beta}(i_0 : i_f)$ is the vector obtained by concatenating the elements i_0 to i_n of the vector $\boldsymbol{\beta}$. Recall that the parameter λ in the Lasso is crucial for accuracy. A classical method to optimally choose this parameter is using cross validation.

However, even after using cross validation, the Lasso tends to induce a high false discovery rate (FDR), i.e., many zero interactions are inferred as non-zeros ones. Formally, the FDR of a inference procedure $\boldsymbol{y} = X\boldsymbol{\beta} + \boldsymbol{z}$, returning the inferred parameters $\hat{\boldsymbol{\beta}}$, is defined as

$$\mathsf{FDR} = \mathbb{E}\left[\frac{\#\{j : \beta_j = 0 \text{ and } j \in \hat{\boldsymbol{\beta}}\}}{\#\{j : j \in \hat{\boldsymbol{\beta}}\} \vee 1}\right].$$

Here $a \vee b = \max\{a, b\}$.

Recently, the so-called Knockoff filter has been proposed as an enhancement to the Lasso algorithm to maintain the FDR below a certain user-defined level $q > 0$, regardless of the value of the coefficients $\boldsymbol{\beta}$ (see [8]). This method works by constructing the so-called "knockoff variables" that mimic the correlation structure found in the real data. The knockoff copy of each variable act as a "control group", allowing to assign a "trust" to each inferred variable. It has been shown this strategy successfully controls the FDR. In our work we used the Matlab package of the Knockoff filter as provided in https://web.stanford.edu/~candes/Knockoffs/package_matlab.html. The validation of the network inference with GLV dynamics is shown in Fig. 4 of the main text.

Blinded inference of interaction strengths by assuming GLV dynamics. Here we show that, if the steady-state samples were collected from a microbial community without GLV dynamics, the inference of interaction strengths by assuming GLV dynamics systematically leads to inference errors.

To illustrate this point, we first generated steady-state samples using Holling Type-II functional response. Then, we applied the GLV-based inference method to the steady-state samples in order to infer the interaction strengths. Supplementary Figure 9 shows that the accuracy (the percentage of correct sign of the inferred interaction strengths compared with the sign of ground truth) of inferred results is very low, even in the absence of noise. This is consistent with the small value of R^2 of fitted hyperplanes, which describe the deviations of samples to those fitted hyperplanes. This suggest that inferring the interactions strengths of a real microbial community without first testing if its dynamics can be described by the GLV model can produce significative errors.

Supplementary Note 6: Real datasets

A synthetic microbial community of eight soil bacteria. In [9] a set of eight heterotrophic soil-dwelling bacterial species were studied for predicting species persistence in different assembled microbial microcosms. The steady-state dataset $\mathcal{X}$ consists of a total of 101 different species combinations: eight solos, 28 duos, 56 trios, eight septets and one octet (Supplementary Figure 10a). Each species combination of cultivation was carried out in duplicate and started from different configurations of initial abundance. We averaged the steady states from different initial conditions.

First we find that R^2 of each fitted hyperplane is less than 0.9 (Supplementary Figure 10b), which indicates that this microbial community could not be properly described by the GLV model. Hence we focus on the inference of interactions types between any two species. To be fair, without considering the eight solos and 28 duos, we analyze the rest steady-state samples. We use both the brute-force algorithm (see Fig. 5 of main text) and the heuristic algorithm (Supplementary Figure 10c,d) to infer the ecological interaction types. In Supplementary Figure 10c, blue (or red) means inhibition (or promotion) effect of species j on species i, respectively. We found that 11 signs were falsely inferred, 5 signs were undetermined by the analyzed steady-state samples. The inferred results are very similar with the brute-force method shown in Fig. 5b of main text. Furthermore, Supplementary Figure 10d shows that once Ψ is larger than a certain value, the accuracy in the inference does not increase any more.

A synthetic community of maize roots with seven bacterial species. (Fig. 6b in the main text). There are in total seven bacterial species (Ecl, Sma, Cpu, Opi, Ppu, Hfr and Cin) in this community [10]. The available steady-state data consists of seven sextets (i.e., data from seven experiments in which six different species grow together) and one septet (i.e., data from one experiment in which the seven species grow together). This leads to a total of eight steady-state samples, see Supplementary Figure 11a.

First, based on our theoretical result showing that in the generalized Lotka-Volterra (GLV) model the steady states that share common species will align into a hyperplane, we concluded that this bacterial community does not follow the GLV dynamics (see Supplementary Figure 11b). Thus, we have to focus on inferring the interaction types, rather than interaction strengths.

Second, only using the seven sextets we inferred the sign-pattern of the Jacobian matrix (Fig. 6a in the main text). Based on the inferred sign of J_{ij}, we can predict how the abundance of species i will change, when we add species j to the community (see results in the Main Text).

A synthetic microbial community of two cross-feeding partners. In this community [11], two non-mating strains of the budding yeast, *Saccharomyces cerevisiae*, were engineered to be deficient in the biosynthesis of one of two essential amino acid tryptophan (Trp) or leucine (Leu), and to overproduce the amino acid required by their partner. It has been demonstrated that these two strains form a community with cross-feeding mutualism, where each strain provides the amino acid needed by its partner. In [11], the authors inoculated monocultures and co-cultures at a range of concentrations of supplemented amino acids in a well-mixed liquid batch. Supplementary Figure 12a-c shows the abundance of the co-cultures and monocultures for the Trp and Leu strains at low, medium and high levels of supplemented amino acids. After seven days cultivation, the abundance of each species approaches its steady state. Note that for each scenario, the experiments inoculate a constant amount of resources at the beginning. Here the type of interaction is defined by comparing the abundance of co-cultures with monocultures at the end of cultivation. As the supply of amino acids increases from low, to medium to high concentrations, the interaction between this pair of strains shifts from obligatory mutualism (Supplementary Figure 12a), to facultative mutualism (Supplementary Figure 12b), and to parasitism (Supplementary Figure 12c), respectively.

We applied our inference method to each scenario. Supplementary Figure 12d-f shows the diagrams of our inference results that are consistent with the empirical observations. For example, in Supplementary Figure 12e,f, the cyan line orthogonal to the red line is very close to the Leu axis,

which indicate the effect of Trp on Leu is very weak. Especially in Supplementary Figure 12f, this promotion effect can be ignored.

A synthetic community of 14 auxotrophic *Escherichia coli* strains. Starting from a prototrophic E. coli derivative MG1655, the authors of [12] generated 14 strains, each containing a gene knockout that lead to an auxotrophic phenotype unable to produce 1 of 14 essential amino acids. By convention, the authors labeled each auxotrophic strain by the amino acid it lacks. For example, the methionine auxotroph Δ*metA* auxotroph is strain M. It was confirmed that the 14 auxotrophs (*C, F, G, H, I, K, L, M, P, R, S, T, W, Y*) show no growth in M9-glucose minimal media after 4 days. Indeed, they grow only when supplemented with the essential amino acid they were not able to produce. This dataset consists of co-cultures of all 91 possible strain pairs from the 14 characterized auxotrophic strains. For each pairwise co-culture, we are able to calculate the total fold growth, i.e., the yield of the community calculated by (total final cell density)/(total initial cell density), as well as the fold growth of each strain. Since these auxotrophic strains cannot grow by themselves, if strain i is able to grow as a co-culture when paired with strain j, and strain i's fold growth is $F_{ij} > 1$, this implies that strain j promotes the growth of strain i, i.e., $J_{ij} > 0$. By contrast, if $F_{ij} < 1$, we cannot conclusively say that $J_{ij} < 0$ because we lack the monoculture data. Therefore, the fold-growth metric can only be used to detect a promotion effect between two strains.

First, we found that R^2 of all fitted hyperplanes are smaller than 0.9, implying that the population dynamics of this microbial community cannot be properly described by the GLV model (Supplementary Figure 13a). Second, we used the heuristic algorithm to infer the interaction types (Supplementary Figure 13b). Note that the complexity of the inference approaches $3^{14} \sim 4 \times 10^6$ if we use the brute-force algorithm. We found that the types of 14 pairwise interactions cannot be determined with the given dataset (marked in gray in Supplementary Figure 13b). Third, we showed the fold growth matrix $F = (F_{ij})$ from experimental observations (Supplementary Figure 13c), with F_{ij} the fold growth of strain i (row) in the co-culture paired with strain j (column). Here we set $F_{ij} \geq 20$ as an indication of promotion effect of strain j on strain i. There are in total 71 promotion interactions with such a large confidence (shown in red, Supplementary Figure 13c). We will use them as the ground truth to check our inference results on promotion effects (i.e., positive signs, shown in red in Supplementary Figure 13b). We found we inferred 13 wrong positive signs (marked as '$\times$' in Supplementary Figure 13c), and missed 5 positive signs (marked as '?' in Supplementary Figure 13c). Therefore, our inference of positive signs has an accuracy of 74.65% (53/71), if we set the fold growth threshold 20 as the indication of promotion effect. We also observed that the accuracy on the inference generally increased by increasing this threshold (Supplementary Figure 13d).

Supplementary Note 7: Relationship to existing notions of inter-taxa interactions

In Assumption 2, we considered that the Jacobian of (S1) determines the interaction types between microbial taxa. This assumption was then used to build our network reconstruction method. Here we discuss how this consideration compares to other existing definitions and notions of "interactions" available in the ecological literature.

In general ecological systems, understanding the interactions between taxa and their strengths is key for developing predictive models and conservation strategies. This has motivated the introduction of several empirical indices for inter-taxa interactions, specially for consumer-prey ecosystems [13, 14]. Let x_1 and x_2 denote the abundances of prey and consumer, respectively. Consider two samples for this ecosystem consisting of an experiment with the prey in isolation $x^{\{1\}} \in \mathbb{R}^2$ —that is, with the consumer or predator deleted— and other with both prey and consumer present $x^{\{1,2\}} \in \mathbb{R}^2$. Here we discuss the four empirical indices as used in [14]:

a) Raw difference: $\mathsf{R} = \frac{x_1^{\{1,2\}} - x_1^{\{1\}}}{x_2^{\{1,2\}}}$.
b) Paine's index: $\mathsf{PI} = \frac{x_1^{\{1,2\}} - x_1^{\{1\}}}{x_1^{\{1\}} x_2^{\{1,2\}}}$.
c) Community importance: $\mathsf{CI} = \frac{x_1^{\{1,2\}} - x_1^{\{1\}}}{x_1^{\{1\}} p}$, where $p = x_2^{\{1,2\}}/(x_1^{\{1,2\}} + x_2^{\{1,2\}})$.
d) Dynamic index: $\mathsf{DI} = \frac{\ln(x_1^{\{1,2\}}/x_1^{\{1\}})}{t\, x_2^{\{1,2\}}}$, where t is time.

Remark 7.

a. All the above indices have identical signs, solely determined by $x_1^{\{1,2\}} - x_1^{\{1\}}$. As shown in Example 3, such sign coincides with one of the possible sign-patterns obtained by applying our reconstruction method for $N = 2$ taxa. More precisely, the above indices coincides with our reconstruction method provided we assume this as prior information. Such prior information can be interpreted as adopting a "convention" for the sign of self-interactions (i.e., a kind of "relative sign-pattern").
b. Compared to the analysis in [14], our reconstruction method provides more general conditions under which the above indices provide the correct sign of the interactions according to a mathematical model.
c. Our reconstruction method also generalizes the application of the above indices to ecosystems with an arbitrary number of taxa, and beyond the consumer-prey interactions.
d. Our reconstruction method provides conditions under which the available steady-state data is informative enough to infer the correct sign of a desired microbial interaction.
e. According to our framework, note there are two different interactions that is possible to infer: $x_1 \to x_2$ and $x_2 \to x_1$. The above indices and discussions are concerning the interaction $x_2 \to x_1$ —that is, the effect of the consumer on the prey. In order to infer the sign of the interaction $x_1 \to x_2$, we need to evaluate $x_2^{\{1,2\}} - x_2^{\{2\}}$. In the case of consumer-prey ecosystem with $N = 2$ taxa, it might be impossible to measure a non-zero $x^{\{2\}}$, since it corresponds to a steady-state abundance of consumers in the absence of prey. In such case, the set $\mathcal{I}_2$ contains only one sample $x^{\{1,2\}}$, and thus the given data is not informative enough to infer this interaction. This argument could explain cases when for N taxa it is impossible to infer some interaction due to the absence of the needed sample, simply because in the absence of some taxa other become extinct.

Supplementary References

[1] Searson, D. P., Leahy, D. E. & Willis, M. J. Gptips: an open source genetic programming toolbox for multigene symbolic regression. In *Proceedings of the International multiconference of engineers and computer scientists*, vol. 1, 77–80 (Citeseer, 2010).

[2] Skalski, G. T. & Gilliam, J. F. Functional responses with predator interference: viable alternatives to the holling type ii model. *Ecology* **82**, 3083–3092 (2001).

[3] Holling, C. S. The functional response of predators to prey density and its role in mimicry and population regulation. *Memoirs of the Entomological Society of Canada* **97**, 5–60 (1965).

[4] Beddington, J. R. Mutual interference between parasites or predators and its effect on searching efficiency. *The Journal of Animal Ecology* 331–340 (1975).

[5] Crowley, P. H. & Martin, E. K. Functional responses and interference within and between year classes of a dragonfly population. *Journal of the North American Benthological Society* **8**, 211–221 (1989).

[6] Hsu, S.-B., Hwang, T.-W. & Kuang, Y. Global dynamics of a predator-prey model with hassell-varley type functional response. *Discrete and Continuous Dynamical Systems. Series B* **10**, 857–871 (2008).

[7] Lang, S. *Complex analysis*, vol. 103 (Springer Science & Business Media, 2013).

[8] Barber, R. F., Candès, E. J. *et al.* Controlling the false discovery rate via knockoffs. *The Annals of Statistics* **43**, 2055–2085 (2015).

[9] Friedman, J., Higgins, L. M. & Gore, J. Community structure follows simple assembly rules in microbial microcosms. *Nature Ecology Evolution* **1**, 0109 (2017).

[10] Niu, B., Paulson, J. N., Zheng, X. & Kolter, R. Simplified and representative bacterial community of maize roots. *Proceedings of the National Academy of Sciences* **114**, E2450–E2459 (2017).

[11] Hoek, T. A. *et al.* Resource availability modulates the cooperative and competitive nature of a microbial cross-feeding mutualism. *PLoS biology* **14**, e1002540 (2016).

[12] Mee, M. T., Collins, J. J., Church, G. M. & Wang, H. H. Syntrophic exchange in synthetic microbial communities. *Proceedings of the National Academy of Sciences* **111**, E2149–E2156 (2014).

[13] Wootton, J. T. Estimates and tests of per capita interaction strength: diet, abundance, and impact of intertidally foraging birds. *Ecological Monographs* **67**, 45–64 (1997).

[14] Berlow, E. L., Navarrete, S. A., Briggs, C. J., Power, M. E. & Menge, B. A. Quantifying variation in the strengths of species interactions. *Ecology* **80**, 2206–2224 (1999).

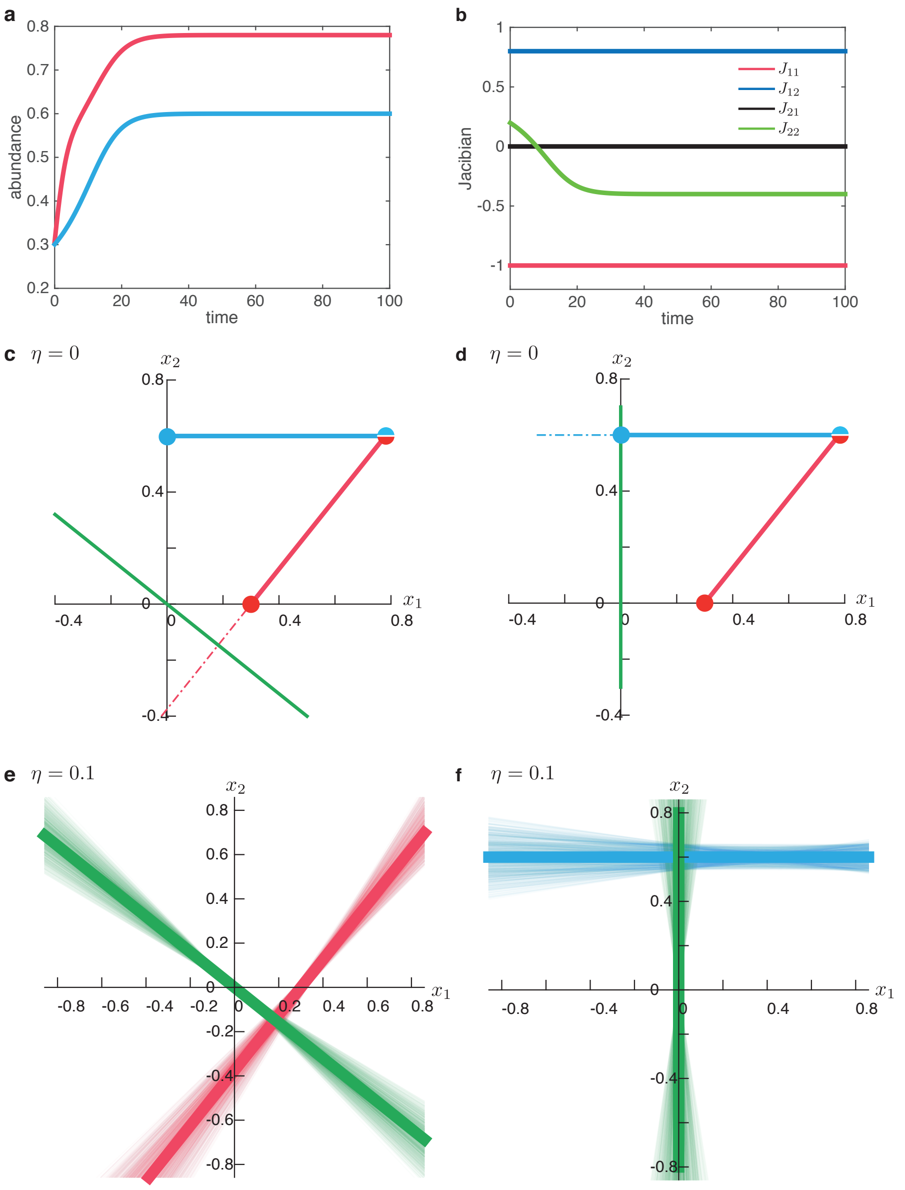

SUPPLEMENTARY FIGURE 1. **Inferring the zero-pattern of the Jacobian matrix. a.** The temporal evolution of the abundance of each species. **b.** The sign of J_{22} is time varying, while the signs of the other elements in the Jacobian matrix are time-invariant. **c.** Inference of J_{12}. According to the position of $\boldsymbol{x}^{\{1,2\}} - \boldsymbol{x}^{\{1\}}$, the green line which is orthogonal to the red line cannot produce a zero entry for $\boldsymbol{J}_1$, implying that $J_{11} \neq 0$ and $J_{12} \neq 0$. This is consistent with the ground truth. **d.** Inference of J_{12}. The green line (orthogonal to the blue line) is aligned with the x_2-axis, indicating that $J_{21} = 0$ and $J_{22} \neq 0$, consistent with the ground truth. **e.** When noise level $\eta = 0.1$, the light green line is orthogonal to the light red line corresponding to the difference of two noisy samples $\boldsymbol{x}^{\{1\}}$ and $\boldsymbol{x}^{\{1,2\}}$. The bold red and green lines correspond to the noiseless case. There are in total 1000 different measurements (replicates). We found that the angles between the green lines and x_1-axis are large enough, letting us conclude that $J_{12} \neq 0$ even if there exists some noise. **f.** When noise level $\eta = 0.1$, the light green line is orthogonal to the light blue line corresponding to the difference of two noisy samples $\boldsymbol{x}^{\{1\}}$ and $\boldsymbol{x}^{\{1,2\}}$. The bold blue and green lines correspond to the noiseless case. There are in total 1000 replicates. Among the 1000 replicates, the light green line is equally distributed to the left and right side of x_2-axis, indicating that the deviation of the light green line from the x_2-axis is likely due to measurement noises. This behavior let us introduce a user-defined cutoff value to judge the zero-pattern of J_{ij} based on the angle between the x_1-axis (or x_2-axis) and green lines.

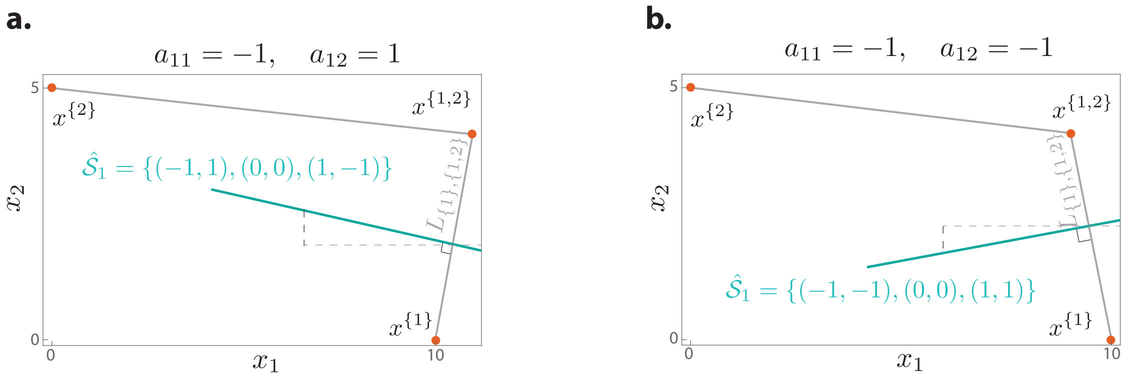

SUPPLEMENTARY FIGURE 2. **Reconstructing** $\mathsf{sign}(\boldsymbol{J}_1)$ **for** $N = 2$ **in Example 3.** For a microbial community of two taxa, its ecological interactions of taxon 1 are shown in the title of each panel. There are three possible steady-state samples, and two of them, $\boldsymbol{x}^{\{1\}}$ and $\boldsymbol{x}^{\{1,2\}}$, share taxon 1. **a.** The green line is orthogonal to $\boldsymbol{x}^{\{1\}} - \boldsymbol{x}^{\{1,2\}}$ and orients three possible orthants, i.e., $\hat{\mathcal{S}}_1 = \{(-1,1),(0,0),(1,-1)\}$. Thus $\mathsf{sign}(\boldsymbol{J}_1)$ belongs to one of the three sign-patterns. **b.** The same procedures for different ecological interactions.

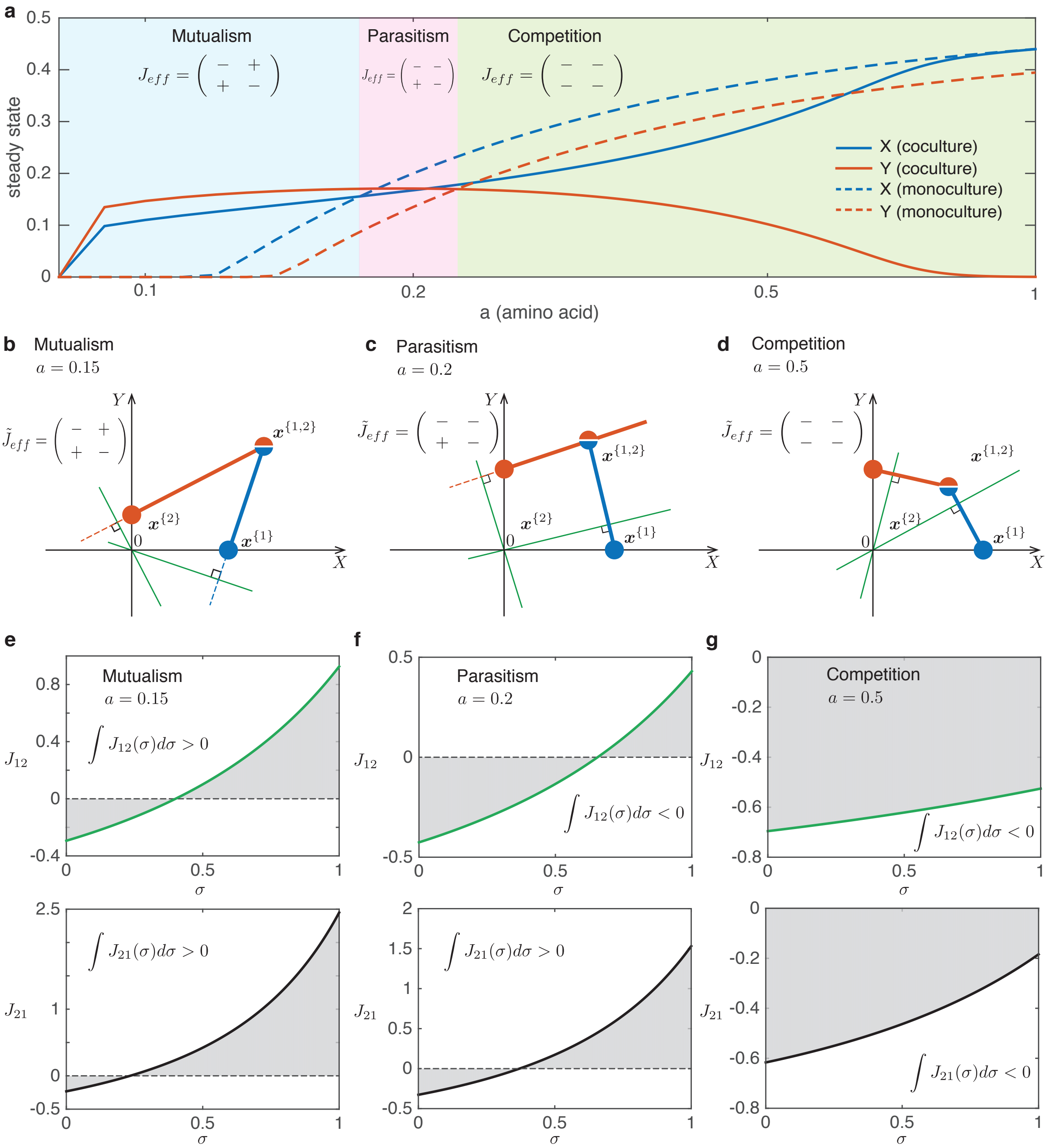

SUPPLEMENTARY FIGURE 3. **In case the sign-pattern of the Jacobian matrix is time varying, the results of our inference can be interpreted as the overall inhibition or promotion impact between different taxa.** Here we consider a toy model of two species X and Y. Each has a per capita growth rate that is modulated by its mutualistic partner as well as the resource amount (denoted as a). The population dynamics model is shown in Eq. (S6), with model parameters $\kappa = 0.12, \delta = 0.5, \beta = 2$. **a.** Three regimes of the interaction types emerge from different resource amount, from mutualism, parasitism to competition. The ground truth of the interaction types is determined by comparing the abundance of coculture (solid lines) with that of monoculture (dashed lines). **b-d.** Diagrams of our inference method under different resource amount. **e-g.** $J_{ij}(\boldsymbol{x}^I + \sigma(\boldsymbol{x}^K - \boldsymbol{x}^I))$ as a function of σ under different resource amount.

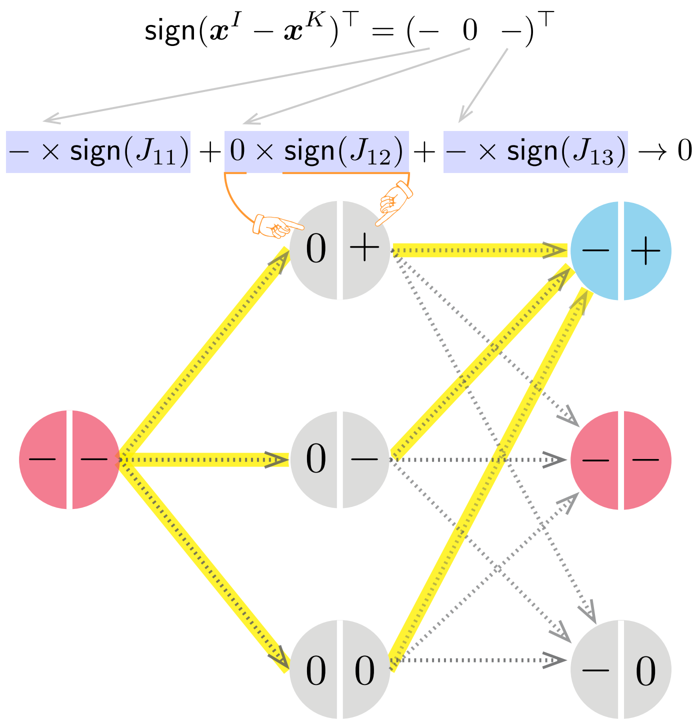

SUPPLEMENTARY FIGURE 4. **Sign-satisfaction graph and its solution for Example 6**. Each node is divided in two parts: the left is an entry of $\mathsf{sign}(\boldsymbol{x}^I - \boldsymbol{x}^K)$ and the right is an entry of $\mathsf{sign}(\boldsymbol{J}_1)$. The color of each node represents the multiplication of the sign of left and right part (red is positive, gray is zero and blue is negative). The yellow paths are the solutions to the sign-satisfaction graph, determining $\mathsf{sign}(\boldsymbol{J}_1)$, i.e., $(-, +, +)$, $(-, -, +)$, and $(-, 0, +)$. Note that since we assume $J_{11} < 0$ as prior information, the first column of the sign-satisfaction graph only has one node.

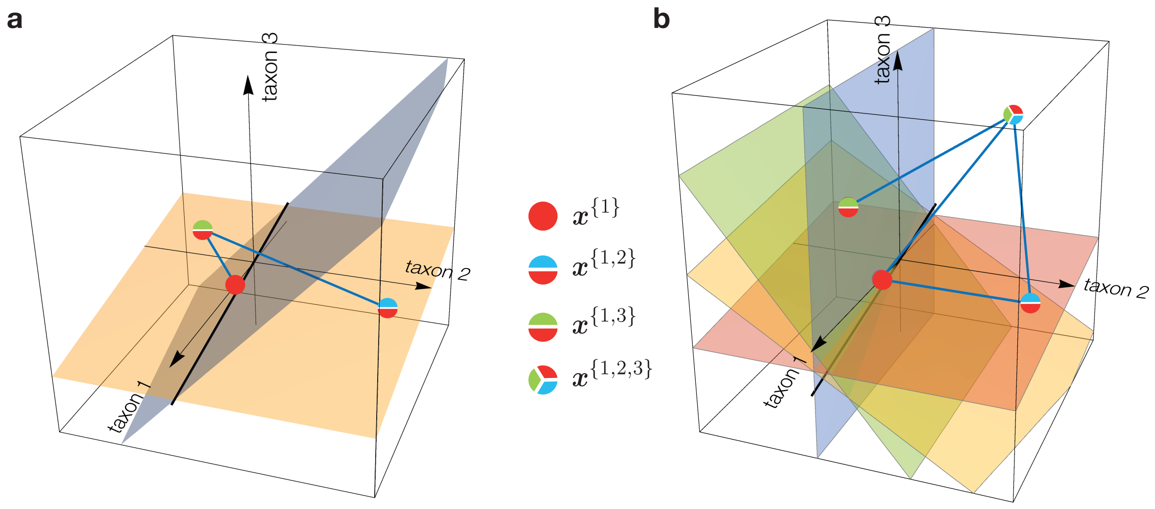

SUPPLEMENTARY FIGURE 5. **Intersections of planes provides a preference sampling in the sign-satisfaction graph. a.** The black line is the intersection of the light orange and blue planes. Each of normal vectors (blue lines) corresponds to the first and fifth column of the matrix M_1 in Example 5, respectively. Note that the orthants to which the intersection line belongs implies that those orthants are at least crossed by two planes. **b.** If there 4 samples sharing taxon 1, we will have a total of $\binom{4}{2} = 6$ planes. Thus, ϕ will be the normalized count of how many of those hyperplane cross the orthants determined by the black intersection line.

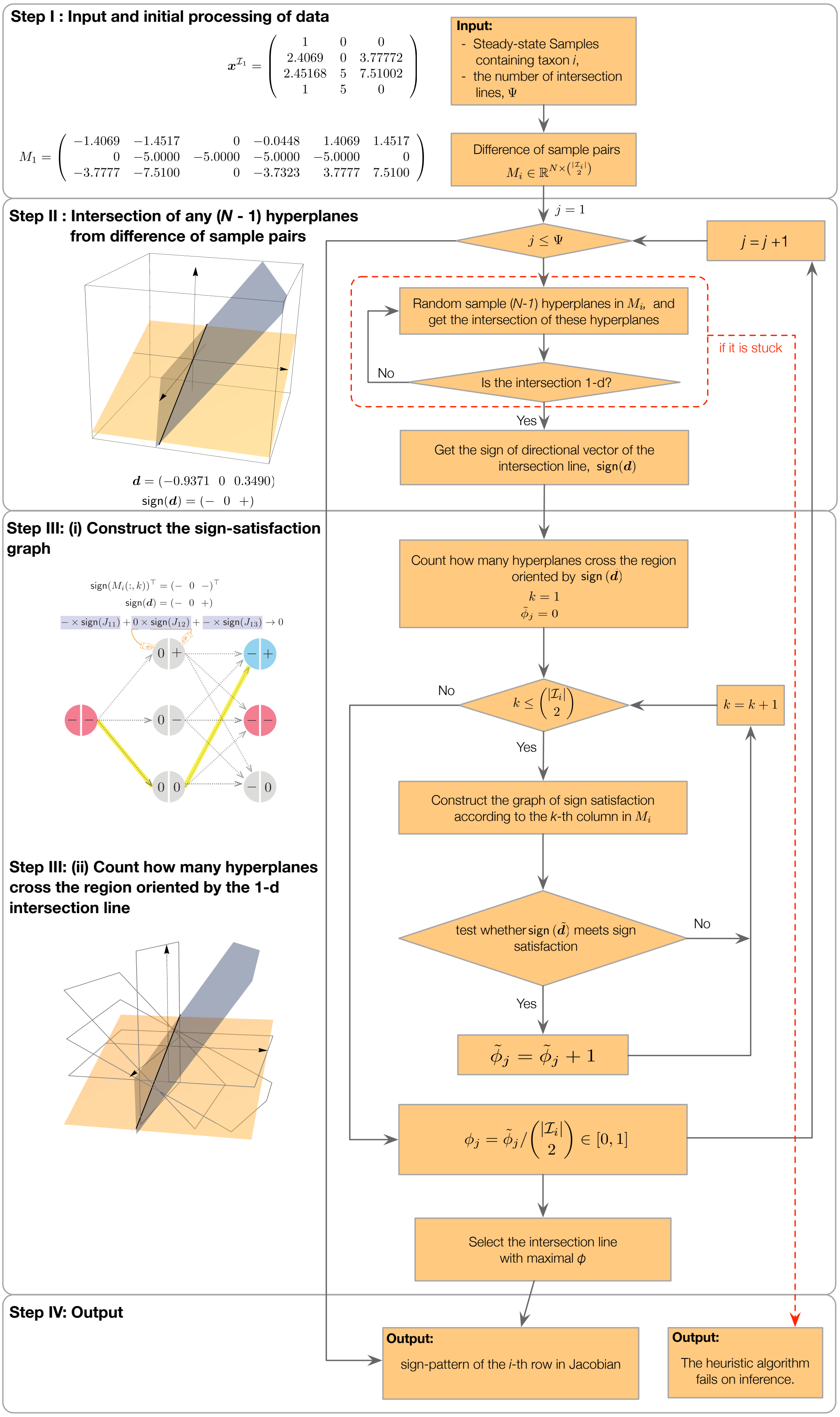

SUPPLEMENTARY FIGURE 6. **Pipeline of the heuristic algorithm for inferring interaction types.**

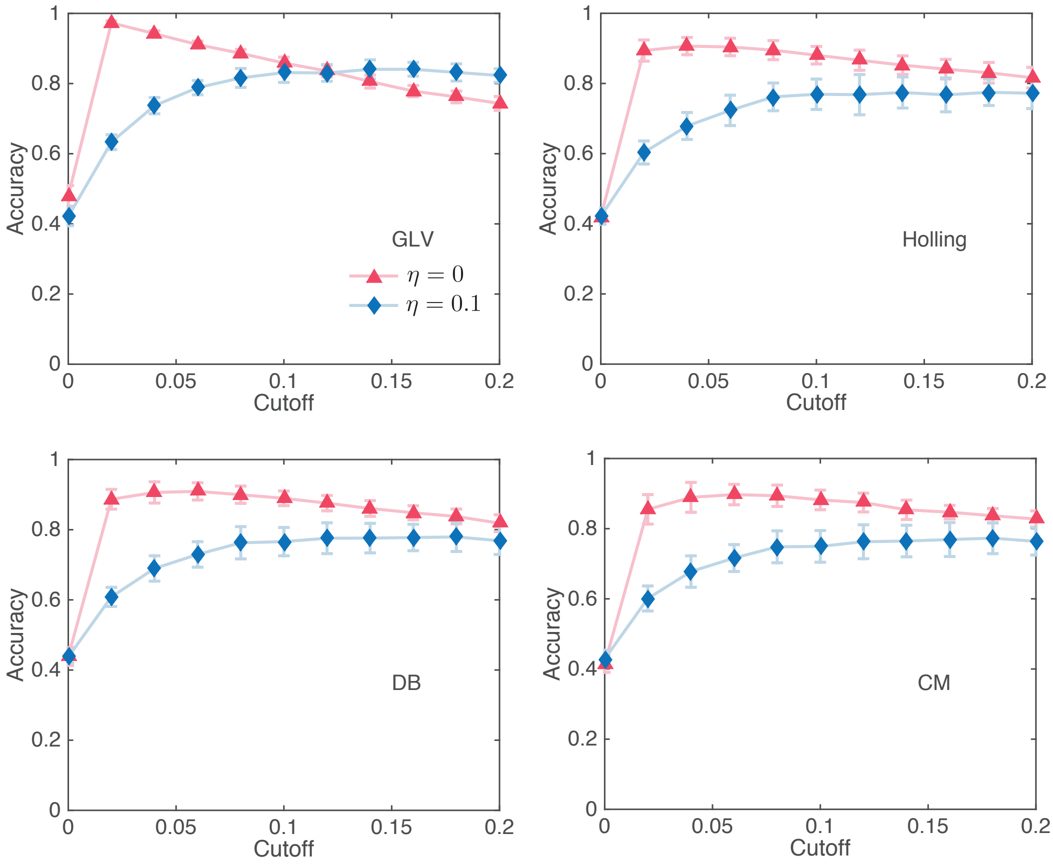

SUPPLEMENTARY FIGURE 7. **The inference of network topology for four different population dynamics models.** Here the ecological network is generated using random network with $N = 20$ taxa and the connectivity equal to 0.4. The simulated steady-state samples are generated using the constants $c_1 = 1, c_2 = c_3 = 0.1$ for Holling, DB and CM. The inference used $\Omega = 5N = 100$ samples and $\Psi = 5N = 100$ intersection lines in the heuristic algorithm. The error bars represent standard deviation for 10 different realizations.

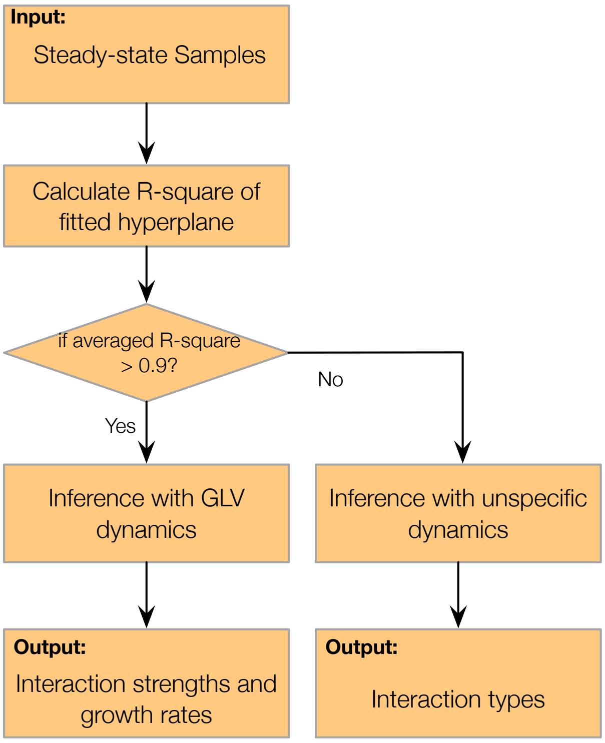

SUPPLEMENTARY FIGURE 8. **Pipeline of the heuristic algorithm for inferring interaction types.**

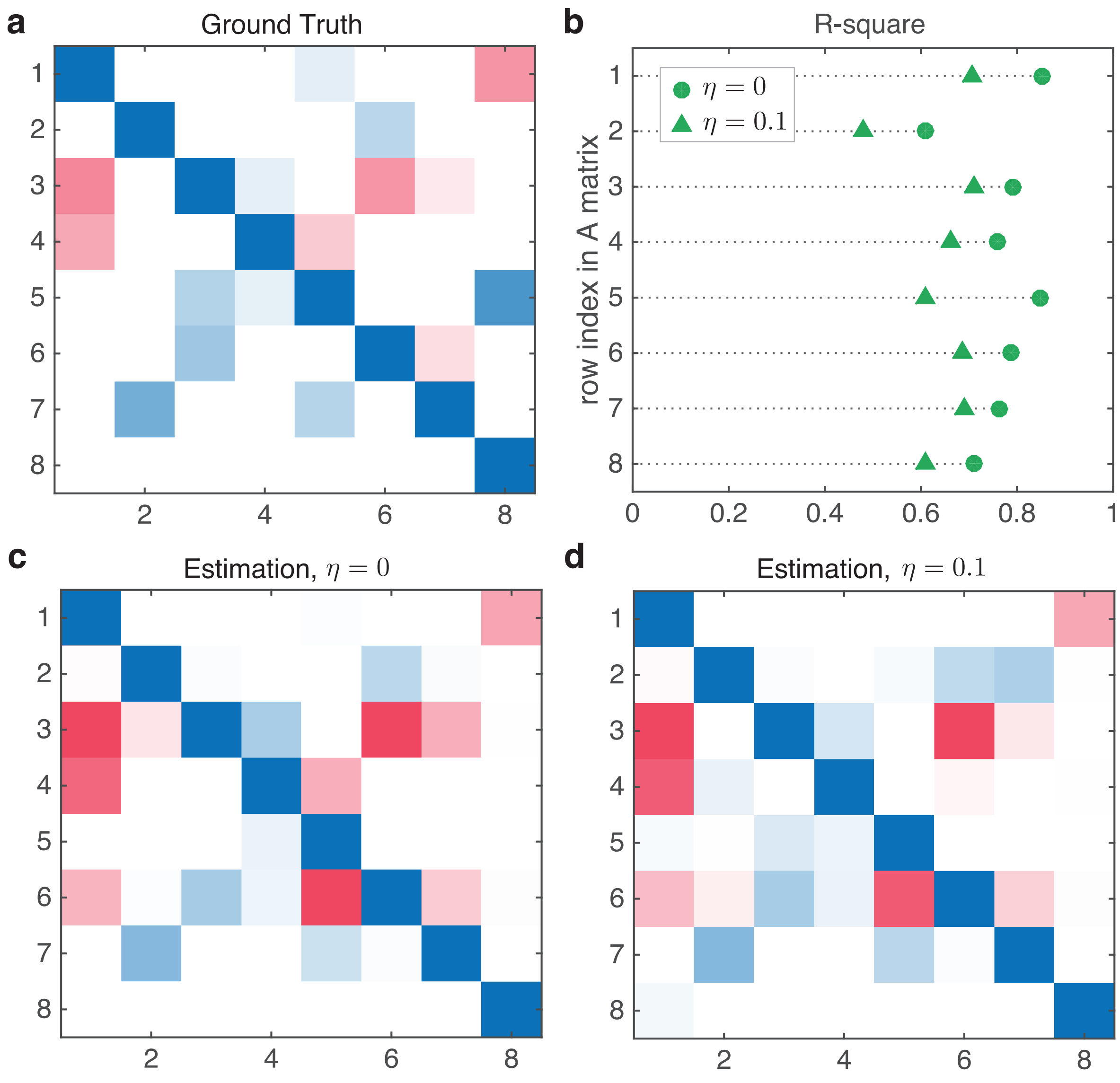

SUPPLEMENTARY FIGURE 9. **Inference assuming GLV dynamics for a microbial community without GLV dynamics. a**. Here we generated the steady states from a microbial community of $N = 8$ taxa with Holling Type-II functional response. The A matrix is shown here. **b**. R^2 of fitted hyperplanes in the noiseless (circle) or noisy (triangle) samples. **c,d**. The inferred A matrix. The accuracy of the inference in the noiseless and noisy cases are 0.7812 and 0.6719, respectively.

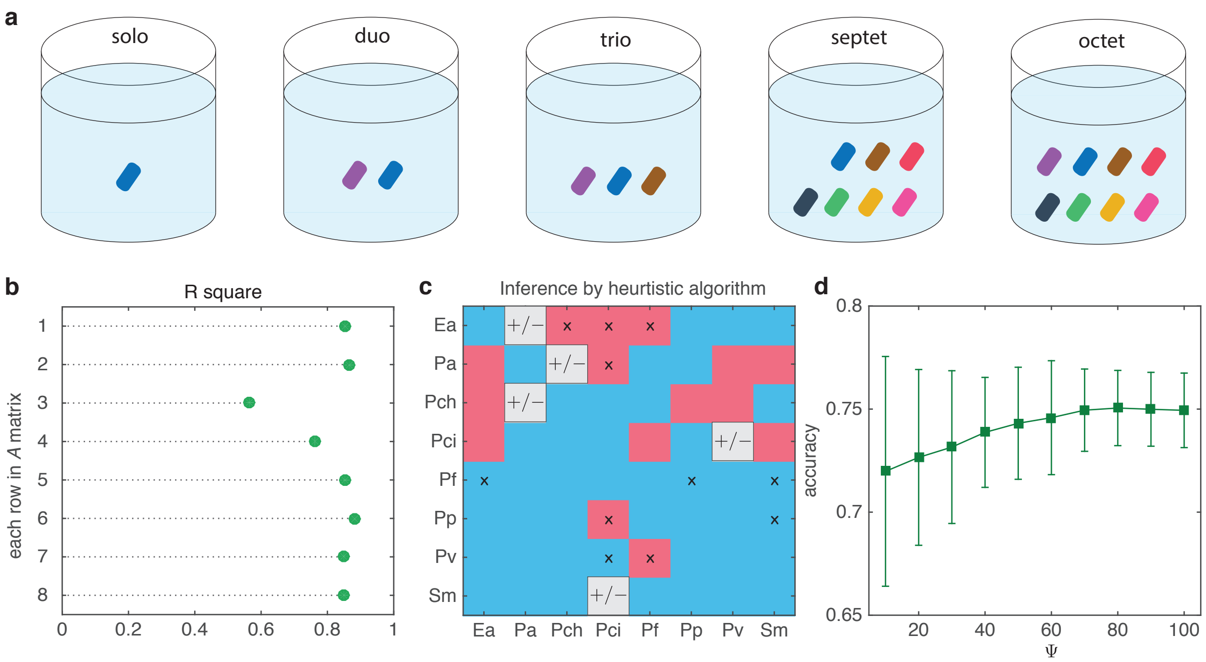

SUPPLEMENTARY FIGURE 10. **Inference of interaction types of a synthetic soil microbial community using our heuristic algorithm. a.** 101 different species combinations: all eight solos, 28 duos, 56 trios, all eight septets, and one octet. **b.** We find that R^2 of all fitted hyperplanes are smaller than 0.9. This suggests that the given samples cannot be properly described by the GLV model, and we should focus on the inference of interaction types. **c.** Inferred interaction types. Compared with the ground truth in Fig. 5a in the main text, there are 11 falsely inferred signs (false labels) and five signs cannot be determined by the given samples. We take the number of intersection line as $\Psi = 50$. **d.** The accuracy as a function of Ψ. Once Ψ is larger than a certain value, the accuracy could not increase any more. The error bar represents standard deviation for 30 different realizations.

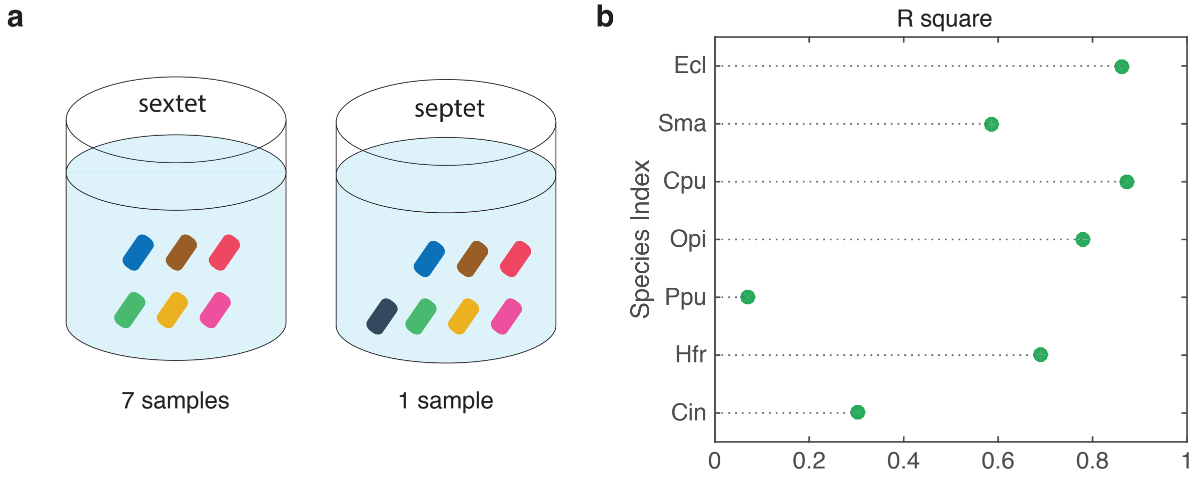

SUPPLEMENTARY FIGURE 11. **Inferring interaction types in a synthetic community of maize roots with seven bacterial species. a.** Eight different species combinations: all seven sextets and one septet. **b.** We find that R^2 of all fitted hyperplanes are smaller than 0.9. This indicates that the given samples cannot be properly described by the GLV model, and we should focus on the inference of interaction types.

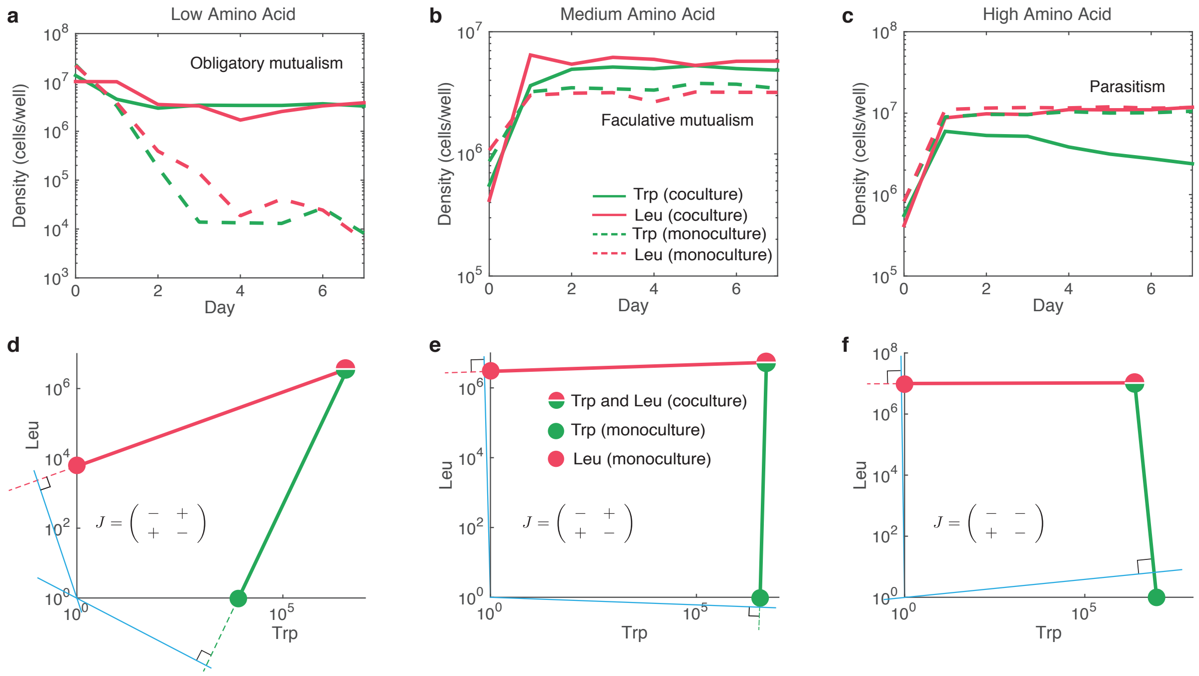

SUPPLEMENTARY FIGURE 12. **Inferring interaction types in a synthetic microbial community of two cross-feeding partners with different amount of resource availability. a-c.** The abundance of the co-cultures (solid line) and monocultures (dashed line) for the Trp (green) and Leu (red) strains with the resources of low, medium and high amino acid. Leu and Trp strains are auxotrophic for each other. However, their co-cultures under different amount of resources exhibit different interaction types. **d-f.** Diagrams of our inference method. The inferred results are consistent with the experimental observations.

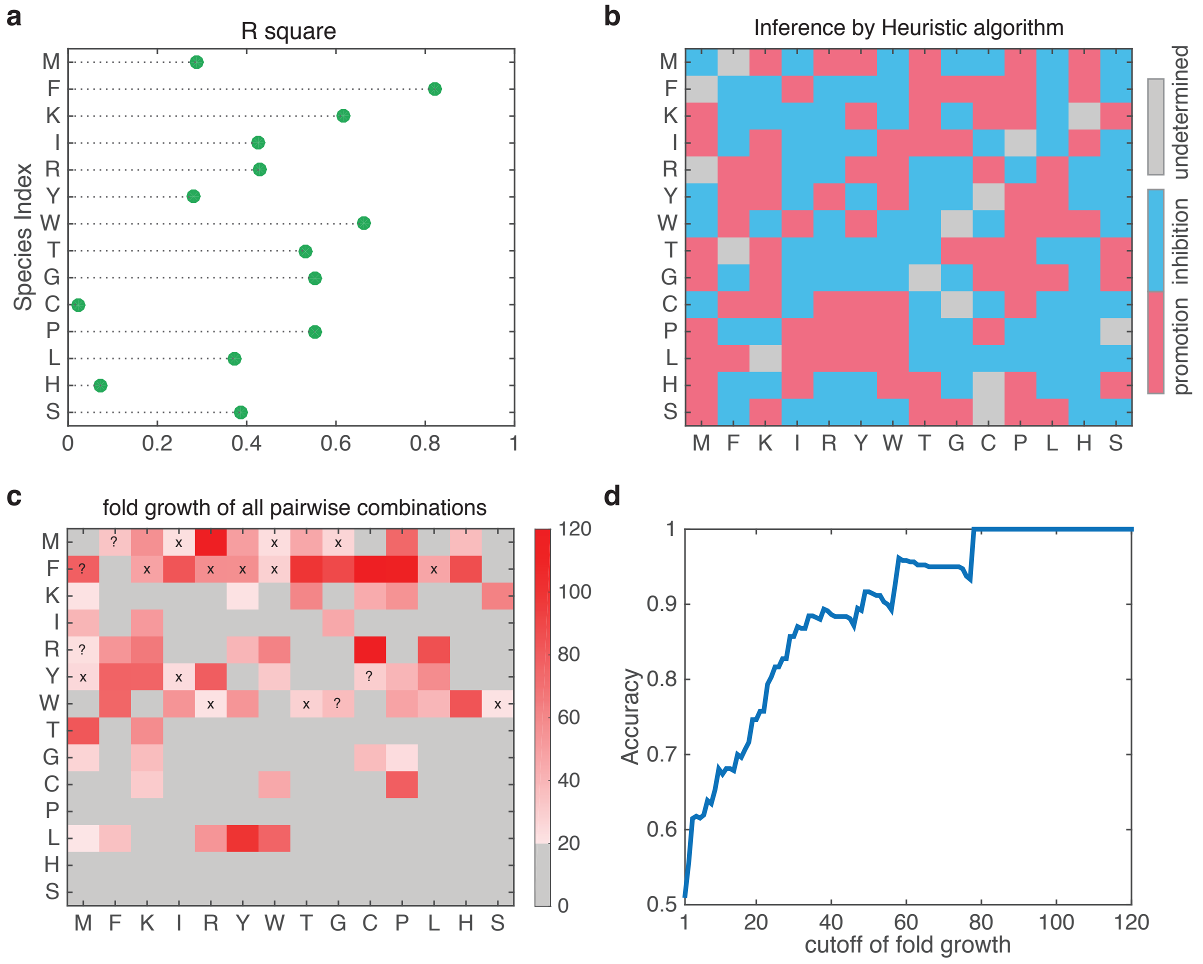

SUPPLEMENTARY FIGURE 13. **Inferring interaction types in a synthetic community of 14 strains each containing a gene knockout that lead to an auxotrophic phenotype of 1 of 14 essential amino acids.** The dataset consists of 91 steady-state samples, each involving a particular pair of the 14 strains. **a.** We find that R^2 of all fitted hyperplanes are smaller than 0.9, suggesting that this community cannot be properly described by the GLV model. **b.** Inferred interaction types by our heuristic algorithm (with 1000 user-defined intersection lines) using only 91 steady-state samples. **c.** The experimentally measured fold growth matrix $F = (F_{ij})$, with F_{ij} the fold growth of strain i (row) in the co-culture paired with strain j (column). We set $F_{ij} \geq 20$ as the indication of promotion effect of strain j on strain i. There are in total 71 promotion interactions with such a large confidence (shown in red). Among them, 53 were correctly inferred, 13 (marked as '$\times$') were not correctly inferred, and 5 (marked as '?') were undetermined by our method, resulting in accuracy 53/71 = 74.65%, at this particular fold growth threshold. **d.** The inference accuracy of promotion effect as a wide range of threshold value of fold growth.
